# Role of bioactive magnetic nanoparticles in the prevention of wound pathogenic biofilm formation using smart nanocomposites

**DOI:** 10.1186/s12951-023-01905-3

**Published:** 2023-05-21

**Authors:** Naeimeh Eghbalifam, Seyed Abbas Shojaosadati, Sameereh Hashemi-Najafabadi

**Affiliations:** 1 Biotechnology Department, Faculty of Chemical Engineering, Tarbiat Modares University, 14155-4838, Tehran, Iran; 2 Biomedical Engineering Department, Faculty of Chemical Engineering, Tarbiat Modares University, Tehran, Iran

**Keywords:** Electrospinning, Iron oxide nanoparticles, Pathogenic biofilm inhibition, Silver nanoparticles, Smart nanocomposites, Wound dressing

## Abstract

**Background:**

Biofilm formation and its resistance to various antibiotics is a serious health problem in the treatment of wound infections. An ideal wound dressing should have characteristics such as protection of wound from microbial infection, suitable porosity (to absorb wound exudates), proper permeability (to maintain wound moisture), nontoxicity, and biocompatibility. Although silver nanoparticles (AgNPs) have been investigated as antimicrobial agents, their limitations in penetrating into the biofilm, affecting their efficiency, have consistently been an area for further research.

**Results:**

Consequently, in this study, the optimal amounts of natural and synthetic polymers combination, along with AgNPs, accompanied by iron oxide nanoparticles (IONPs), were utilized to fabricate a smart bionanocomposite that meets all the requirements of an ideal wound dressing. Superparamagnetic IONPs (with the average size of 11.8 nm) were synthesized through co-precipitation method using oleic acid to improve their stability. It was found that the addition of IONPs to bionanocomposites had a synergistic effect on their antibacterial and antibiofilm properties. Cytotoxicity assay results showed that nanoparticles does not considerably affect eukaryotic cells compared to prokaryotic cells. Based on the images obtained by confocal laser scanning microscopy (CLSM), significant AgNPs release was observed when an external magnetic field (EMF) was applied to the bionanocomposites loaded with IONPs, which increased the antibacterial activity and inhibited the formation of biofilm significantly.

**Conclusion:**

These finding indicated that the nanocomposite recommended can have an efficient properties for the management of wounds through prevention and treatment of antibiotic-resistant biofilm.

**Graphical Abstract:**

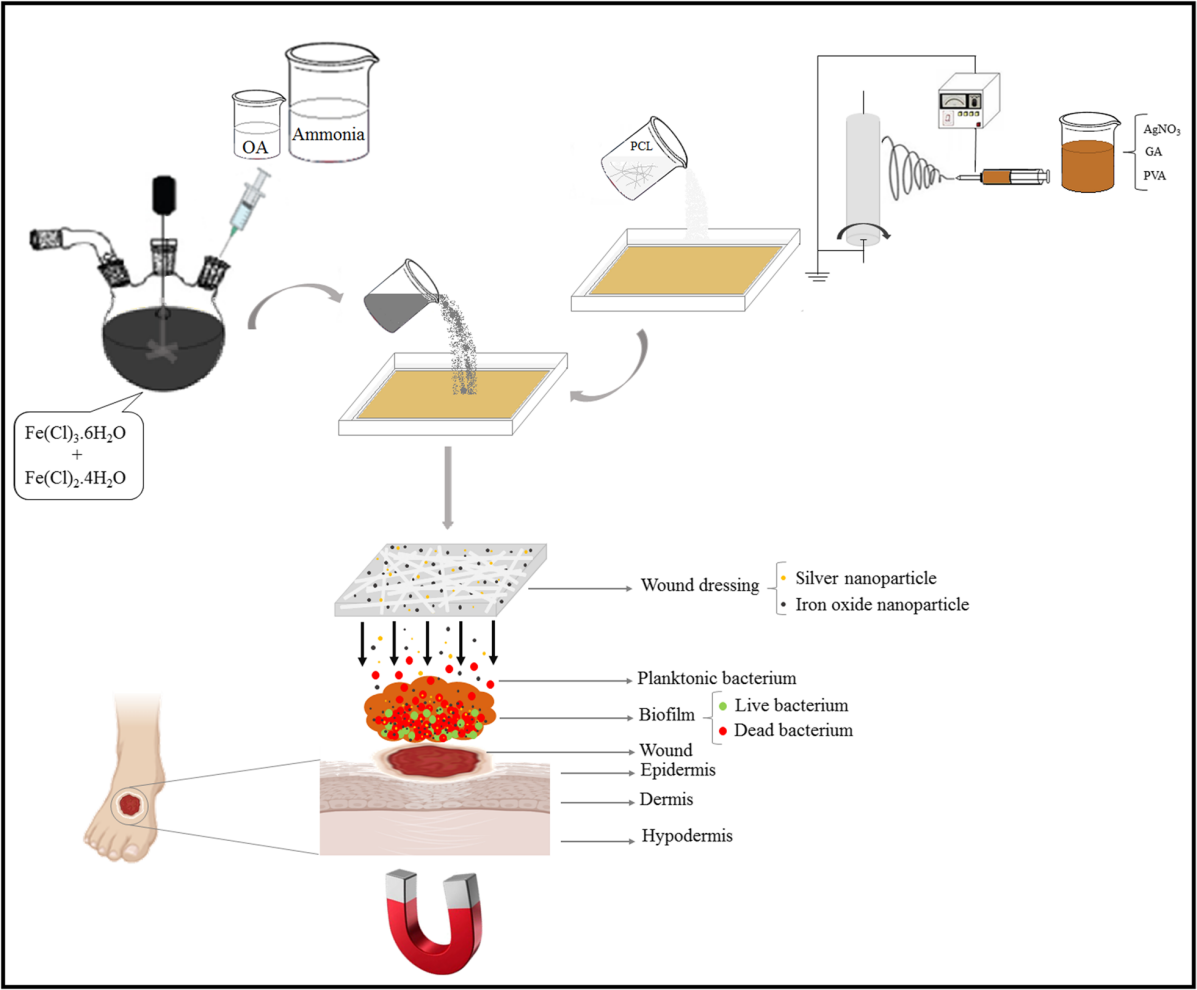

## Introduction

Bacterial infections, as a serious health problem, are among the leading causes of death, especially in skin wound treatment [1–3]. It has been shown that antimicrobial therapy fails due to the increased bacterial resistance to various antibiotics [4–6]. In chronic infections, complete inhibition of bacterial growth via antibiotics is not possible since the growth state changes from planktonic to biofilm [7–13]. Biofilm is a community of microorganisms attached to a particular surface and is embedded in a self-made extracellular polymeric substance (EPS) [9, 12, 14–16]. The EPS matrix reduces the penetration of antibiotics to reach the microorganisms within the biofilm. This is why biofilms are highly resistant to most antibiotics (up to 1000 times more than planktonic cells) [8, 10]. Additionally, since the polymer matrix keeps the nutrients, biofilms are more likely to survive, and this is the reason antibiotics are not able to destroy the biofilm cells [17]. Therefore, biofilm formation is one of the most common challenges in the wound healing process, affecting the treatment time, cost, and failure rate [18, 19]. Thus, it is necessary to develop new approaches to inhibit biofilm formation, treat bacterial infections, and be less toxic to patients.[20, 21].

The strong antibacterial activity of the AgNPs makes them attractive agents for preventing the evolution of antibiotic resistance. They can attack different sites of the cells which are vital to physiological functions (e.g., the cell wall, DNA/RNA synthesis, and electron transport) [12, 22–24]. However, these nanoparticles have some drawbacks including cytotoxicity [10, 24, 25], easy oxidation [12, 26], aggregation [7, 24, 27], and limited penetration into tissues and biofilms [28, 29].

Some solutions have been introduced to overcome these issues. For instance, integration of AgNPs with biopolymers can prevent them from aggregation and oxidation [30]. In addition, biopolymers can provide other benefits such as the ability to absorb wound exudates, non-toxicity, and biocompatibility, which are essential qualities of an ideal wound dressing [5]. Among biopolymers, gums have a high absorbance capacity, which makes them promising materials for this application [31, 32]. Gum Arabic (GA) is an inexpensive material that possesses interesting properties, such as hydrophilicity, biocompatibility, and biodegradability. It can be used in the green synthesis of AgNPs with no additional reducing agents [32]. Moreover, polyvinyl alcohol (PVA) is a synthetic, hydrophilic, and non-toxic polymer with excellent wound dressing properties that can improve gum Arabic’s electrospinability.

On the other hand, polycaprolacton (PCL) is a synthetic biodegradable polyester that has suitable mechanical properties and can be applied to biomedical applications. It increases the structural strength of composites in humid environments.

The problem of low penetration and consequently poor antibacterial efficacy of AgNPs against biofilm infections can be compensated by IONPs since they are responsive to EMF [33, 34]. In particular, magnetite nanoparticles (Fe_3_O_4_) are among the most popular nanoparticles in biomedical applications due to their unique properties, including superparamagnetism and biocompatibility [6, 35, 36]. Therefore, these binary systems combine the benefits of both nanoparticle types, and by integrating silver and iron oxide nanoparticles in polymers with optimum composition, a smart nanofiber composite can be synthesized that provides all the features an ideal wound dressing needs.

In our previous study, GA/PVA/PCL nanocomposites were prepared using a combination of the electrospinning and the coating methods, resulting in the optimum composition in the nanocomposite based on the porosity, water absorption, and water vapor permeability, but its effectiveness on inhibiting biofilm formation and increasing antibacterial activity was not explored. Consequently, the present study aims to develop a desirable fiber nanocomposite (containing silver and iron oxide nanoparticles) as a suitable wound dressing, that will provide a synergistic effect to inhibit the formation of antibiotic-resistant biofilms. The synthesized IONPs, characterized using TEM, DLS, XRD, FTIR, TGA, and VSM analyses, were added to silver-containing nanocomposites and their performance was evaluated through various assays. Nanocomposites with different concentrations of IONPs were investigated for their magnetic properties, and their antimicrobial activity was determined by measuring the radius of inhibition zone. Additionally, cytocompatibility was assessed by cytotoxicity and proliferation studies on mouse embryonic fibroblast cells. As a final step, the biofilm inhibitory abilities of the nanocomposites were evaluated against two common wound pathogen biofilm: *Staphylococcus aureus* and *Pseudomonas aeruginosa*.

## Materials and methods

### Materials

PVA, Mw: 85000–124000 g/mol, PCL, Mw: ~ 80000 g/mol and 3-(4,5-dimethylthiazol-2- yl)2,5-diphenyltetrazolium bromide (MTT) were purchased from Sigma-Aldrich (USA). GA, dimethylformamide (DMF, 99.80%), chloroform (99.00–99.40%), silver nitrate (99.80%), ferrous chloride tetrahydrate (FeCl_2_.4H_2_O), ferric chloride heptahydrate (FeCl_3_.7H_2_O), oleic acid, and ammonia solution (32%) were supplied from Merck (Germany). All chemicals and solvents were of analytical grade and used without any further purification. *Staphylococcus aureus* (IBRC-M 10917) and *Pseudomonas aeruginosa* (ATCC 27853) were obtained from the Iranian Biological Resource Center (IBRC) and the American Type Culture Collection (ATCC), respectively. Mouse embryonic fibroblast (MEF) cells (RSCB0182) were obtained from the Royan Institute (Iran) for Stem Cell Biology and Technology.

### Samples preparation

#### Preparation of electrospinning solutions

Based on the optimal composition obtained in our previous study [32], 348 mg GA and 366 mg PVA were dissolved in deionized water (3 mL) and dimethylformamide (DMF, 3 mL, at 90 °C), respectively, under magnetic stirring until homogenous solutions were obtained. Then, silver nitrate was added to the GA solution. The concentration of silver nitrate in different nanocomposites were 0 (samples 1, 5, 9, and 13), 0.096% (samples 2, 6, 10, and 14), 0.96% (samples 3, 7, 11, and 15), and 1.92% (samples 4, 8, 12 and 16), respectively. In the process of dissolving the silver nitrate, AgNPs were synthesized, and the solution gradually darkened in color. GA-Ag + and PVA solutions were mixed overnight after the silver nitrate was completely dissolved.

#### Fabrication of GA/PVA/Ag fibrous nanocomposite

A 10 mL syringe was filled with the prepared solution, and the electrospinning process was used to produce nanofibers on an aluminum foil. All of the electrospinning parameters were chosen based on our previous research (feeding rate: 0.5 mL/h, applied voltage: 18 kV, tip-to-collector distance: 150 mm, rotation speed of collector: 500 rpm, electrospinning time: 12 h) [32].

#### Coating of the fibrous nanocomposite

186 mg PCL was dissolved in chloroform and the resultant nanocomposite from the last section was immersed in this solution, and then the solvent was evaporated with a lab oven (50 °C). After that, the PCL-coated sheet was peeled off from the aluminum foil [32].

#### Synthesis of IONPs coated with oleic acid

Oleic acid-coated Fe_3_O_4_ nanoparticles were synthesized by the chemical co-precipitation method. Oleic acid was used to improve the stability and consequently better performance of the IONPs. FeCl_2_·4H_2_O (199 mg) and FeCl_3_·6H_2_O (540 mg) as precursors were dissolved in 60 mL of deionized water (ambient temperature). The mixture was exposed to N_2_ in a closed system to avoid ferrous ion oxidation. While stirring, 100 μL of oleic acid as the surfactant and 7 mL of ammonia solution (32%) were added to the reaction mixture immediately. The temperature was raised to 60 °C, and oleic acid was added twice more at 5-min intervals. After 30 min, IONPs were collected by the use of a magnet and then washed with water three times [37].

#### Preparation of Ag/IO nanocomposites

In order to fabricate Ag/IO nanocomposites, IONPs were added to the nanocomposites via an adsorption procedure. Desired concentrations of IONPs (based on the experimental design pre-tests data) were dispersed in isopropanol through ultrasonication (30 min). Afterward, the fibrous nanocomposites (containing different concentrations of the AgNPs, determined by the pre-tests of the experimental design) were immersed in the suspension for 1 h using an orbital shaker.

### Experimental design

A full factorial design of experiments was used to explore the effects of two variables including the AgNPs’ concentration (in 4 levels) and the IONPs’ concentration (in 4 levels). All experiments were carried out in triplicate at least, and the results are expressed as: mean ± SD. Statistical analysis was performed using the one-way ANOVA followed by the Tukey HSD test for post hoc comparisons. P-values less than 0.05 (p < 0.05) were regarded as significant.

#### Levels of the variables

Based on the results of the pre-tests, the levels of each variable were determined (data not shown). Thus, 0, 0.05, 0.5, 1%, and 0, 5, 10, 15%, were chosen for the concentrations of the silver nanoparticles and the IONPs (16 samples).

**Fig. 1:**
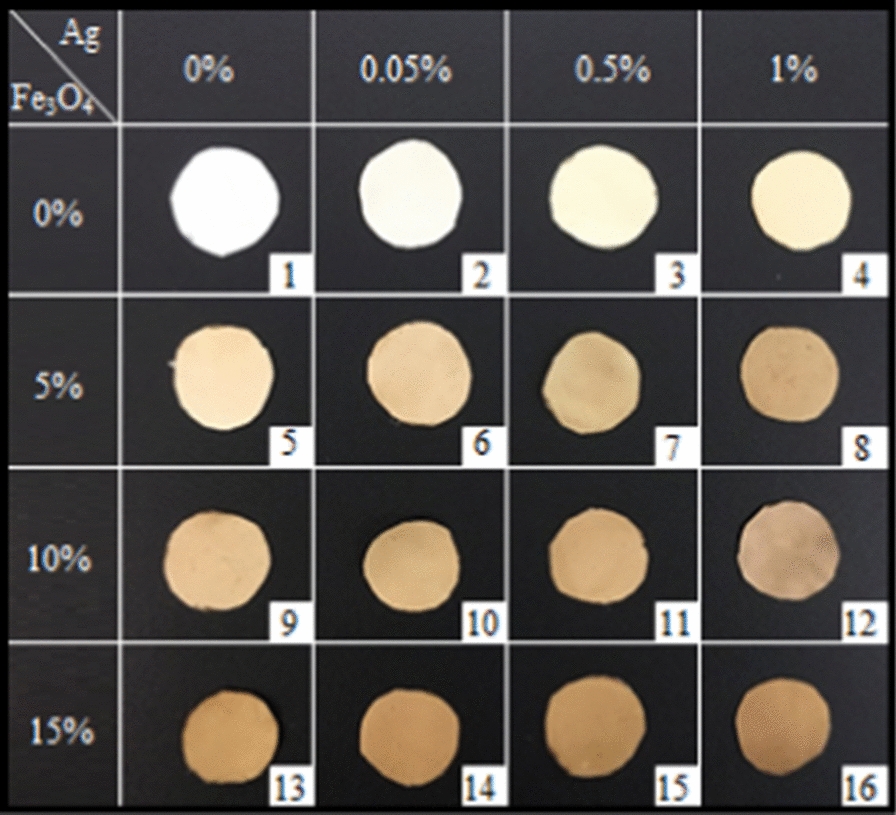
The color of the nanocomposites with different concentrations of AgNPs (0, 0.05, 0.5, and 1%) and IONPs (0, 5, 10, and 15%), samples of 1–16

The images of the prepared samples are given in Fig. 1. The color of the nanoparticles-free sample is white, but changes to yellow when AgNPs are added. The presence of the IONPs in the nanocomposites changes their color into brown. A higher nanoparticle concentration results in a darker sample color.

#### Evaluation of nanocomposite

The effects of the variables on the characteristics and the performance of the resultant nanocomposite were investigated in various ways as follows:

A. Antibacterial assay

#### Agar diffusion method

The antibacterial activity of the nanocomposites against *S. aureus*, *methicillin resistant Staphylococcus aureus (MRSA)*, *P. aeruginosa*, *E. coli* and *C. albicans* was evaluated using the agar diffusion test. The surface of each agar plate was inoculated by swabbing with 0.5 McFarland turbidity standard microbial suspensions (0.5 McFarland turbidity standard provided optical density between 0.08 and 0.1 at 600 nm, roughly equivalent to 1.5 × 10^8^ CFU/mL). Samples were placed on the surface of the inoculated plates. After 24 h incubation at 37 °C, the inhibition zone formed around each sample was measured in five directions, and the average diameter was determined [38].

#### Plate count method (colony forming efficiency)

The two bacteria (*S. aureus* and *P.* aeruginosa) were cultivated in 10 mL of the bacterial suspension in nutrient broth (0.5 McFarland concentration standard) and one piece of each nanocomposite (1 cm × 1 cm) was immersed in bacterial suspension in each tube. The tubes were incubated for 24 h at 37 °C. Then, 100 μL of the content of each tube was taken out and serially diluted (up to seven times) and spread on nutrient agar. The plates were incubated for 24 h at 37 °C. The number of bacterial colonies was counted and the average count of the three plates was determined. Finally, bacterial growth inhibition was calculated as follows:

$$\mathrm{Bacterial\, growth\, inhibition }(\mathrm{\%}) =\frac{(A-B)}{A} \times 100$$

where A & B are the numbers of bacterial colonies (CFU/mL) of control (sample 1) and sample plates, respectively.

B. Measurement of reactive oxygen species (ROS) generation

The ROS generation was determined according to the previously reported protocol using 2ʹ,7ʹ-dichlorofluorescein diacetate (DCFH-DA, Sigma, Germany, D6883) with slight modification [33, 39]. Bacterial cells (10^6^ CFU/mL) were treated with the nanocomposites (1 × 1 cm^2^) at 37 °C for 18 h. Then the growth media was removed and after addition of 2 mL PBS, cells were centrifuged at 1500 rpm for 5 min at 4 °C. The bacteria were collected and resuspended in 1 mL PBS and incubated with 2 µL DCFH-DA in darkness at 37 °C for 45 min. Then 1 mL PBS was added to each sample and they were centrifuged for 5 min at 1500 rpm to remove the residual DCFH-DA solution and the pellets were resuspended in 0.5 mL PBS. Finally, 3 µL of propidium iodide was added to each sample and the ROS production was analyzed using BD FACS Calibur flow cytometer (BD biosciences, San Jose, CA, USA). The bacteria in presence of nanoparticles-free nanocomposites were taken as control.

C. In vitro cytotoxicity

In vitro cytotoxicity effect of the nanocomposites was assessed with the 3-(4,5-dimethylthiazol-2-yl)-2,5-diphenyltetrazolium bromide (MTT) assay on the fibroblast and macrophage cells. Within each sample (1 cm^2^), the MEF cells at a density of 10^4^ cells/well were seeded in Dulbecco’s modified Eagle’s medium (DMEM)/high glucose supplemented with 15% fetal bovine serum (FBS) and 1% penicillin/streptomycin in a 48-well culture plate, and incubated in a humidified atmosphere, 5% CO_2_ at 37 °C. The control well contained only the culture medium, without any nanocomposite (assuming 100% cell viability) [40]. Five replicates were performed to determine the cell viability of each sample at days 1, 3, 5, and 7 by the MTT assay. After each prearranged incubation time, 150 μL fresh medium containing the MTT solution was replaced with the old medium and the plate was incubated for 3 h at 37 °C to let the living cells produce formazan. After that, the medium including residual MTT was removed, dimethyl sulfoxide (DMSO) was added to each well, and 100 μL of the new medium was transferred to a 96-well plate. Finally, the optical density (OD) of the samples was measured by ELISA (enzyme-linked immunosorbent assay) reader at a wavelength of 570 nm. The cell viability percentage was determined by using the following equation [41]:

$$Cell \,viability \left(\%\right)=\frac{{Abs}_{570} (Treated\, cells)}{{Abs}_{570} (Control \,cells)}\times 100$$

D. Biofilm inhibition

To evaluate the antibiofilm activity of the prepared samples, *Staphylococcus aureus* and *Pseudomonas aeruginosa* bacteria were used. The nanocomposites were sterilized with ethanol (99%) and placed in 96-well plates. Then, 180 µL sterile culture medium (nutrient broth) and 20 µL bacteria solution (10^6^ CFU/mL) were added to the wells. Plates were incubated at 37 °C for 24 h to allow the formation of biofilms. The excessive medium was then slowly removed, and the wells were washed with autoclaved PBS and stained with 100 µL crystal violet (0.1%). After 15–30 min, the wells were rinsed again with autoclaved PBS three times and then dried. In the next step, 100 µL ethanol (99%) was added to each well to dissolve the adsorbed crystal violet by the biofilms. The same procedure was also used for the control wells, except for the omission of the nanocomposites. [42–44].

The optical density (OD) of the mixed solution was measured at 595 nm using ELISA reader. The biofilm Inhibition was calculated using the following equation:

$$Biofilm \,Inhibition \left(\%\right)=(\frac{{OD}_{Control}-{OD}_{sample}}{{OD}_{Control}})\times 100\mathrm{\%}$$

For visual inspection of the biofilm inhibition by the nanocomposites and the effect of the EMF application, Confocal Laser Scanning Microscopy (CLSM) was used. Sterile samples were placed in 12-well plates, and 1.5 mL of sterile culture medium (nutrient broth) and 0.5 mL of bacteria solution (1.5 × 10^8^ CFU/mL) were added into the wells. Then, the plates were incubated at 37 °C for 48 h to form biofilm. After that, wells were rinsed three times with PBS, stained with SYTO9 (2 µM) and propidium iodide (PI) (4 µM) incubated for 15 min, and analyzed by CLSM (Zeiss LSM700). The wavelengths of 488 and 525 nm were used as excitation and emission wavelengths for the detection of SYTO9, respectively. Propidium iodide was excited at 520 nm, and its emission was measured at 620 nm. The wells without nanocomposite were considered to be the control groups [7, 33].

### Characterization of the samples and their components

The morphology of IONPs was studied using a Philips EM 208S transmission electron microscopy (TEM). To prepare samples for TEM analysis, iron oxide nanoparticle solution was dropped on the cooper grid and dried at ambient temperature. Dynamic light scattering (DLS) analysis was performed using Particle Metrix GmbH (Germany) to determine the hydrodynamic diameter (D_H_) and the particle size distribution of IONPs. X-ray diffraction (XRD) analysis was carried out using X'Pert MPD (Philips, Netherlands) with Co-kα radiation (where λ = 1.54056 Å and the Bragg's angel, 2θ, in the range of 5–80°). Fourier-transform infrared (FTIR) spectra were recorded using a Perkin–Elmer ATR–FTIR spectrometer spectrum 400. The wave number range and the resolution were 400–4000 cm^−1^, and 2 cm^−1^, respectively. Thermo-gravimetric analysis (TGA) and the corresponding derivative (dTGA) were performed using a Dupont 951 in the temperature range of 25–700 °C in an N_2_ atmosphere to confirm the surface modification of IONPs by oleic acid. The magnetic properties of the IONPs and the nanocomposites were investigated using MDKFT (Danesh Pajouh Kashan Co., Iran) vibrating sample magnetometer (VSM) with a variation of the applied field between − 12000 and 12000 Oe at ambient temperature. To study the morphology and distribution of silver and iron oxide nanoparticles in the nanocomposites, field-emission scanning electron microscopy (MIRA 3, TESCAN, Czech Republic), energy-dispersive x-ray spectroscopy (EDX), and element map were used.

## Results and discussion

### IONPs characterization

Ammonia solution was added to the mixture of the iron salts (in bright yellow) and its color turned to black, resulting in the generation of iron (III) & iron (II) hydroxides because of the hydrolysis of Fe^3+^ and Fe^2+^, respectively. In addition, iron (III) hydroxide was converted to another compound (FeOOH) that reacted with Fe(OH)_2_ to produce Fe_3_O_4_. Fe^2+^ to Fe^3+^ molar ratio was 1:2 to obtain high efficiency in magnetite production and to prevent the oxidation of Fe^2+^ to Fe^3+^. General reactions in the process of Fe_3_O_4_ formation are listed below (Eqs. (1–4)) [45].

1 $${Fe}^{3+}\left(aq\right)+3{OH}^{-}(aq)\to Fe(OH{)}_{3}(aq)$$

2 $${Fe\left(OH\right)}_{3}\left(aq\right)\to FeOOH\left(aq\right)+{H}_{2}O\left(l\right)$$

3 $$F{e}^{2+}\left(aq\right)+2{OH}^{-}(aq)\to {Fe\left(OH\right)}_{2}(aq)$$

4 $$2FeOOH\left(aq\right)+Fe(OH{)}_{2}\left(aq\right)\to {Fe}_{3}{O}_{4}\left(s\right)+2{H}_{2}O(l)$$

**Fig. 2:**
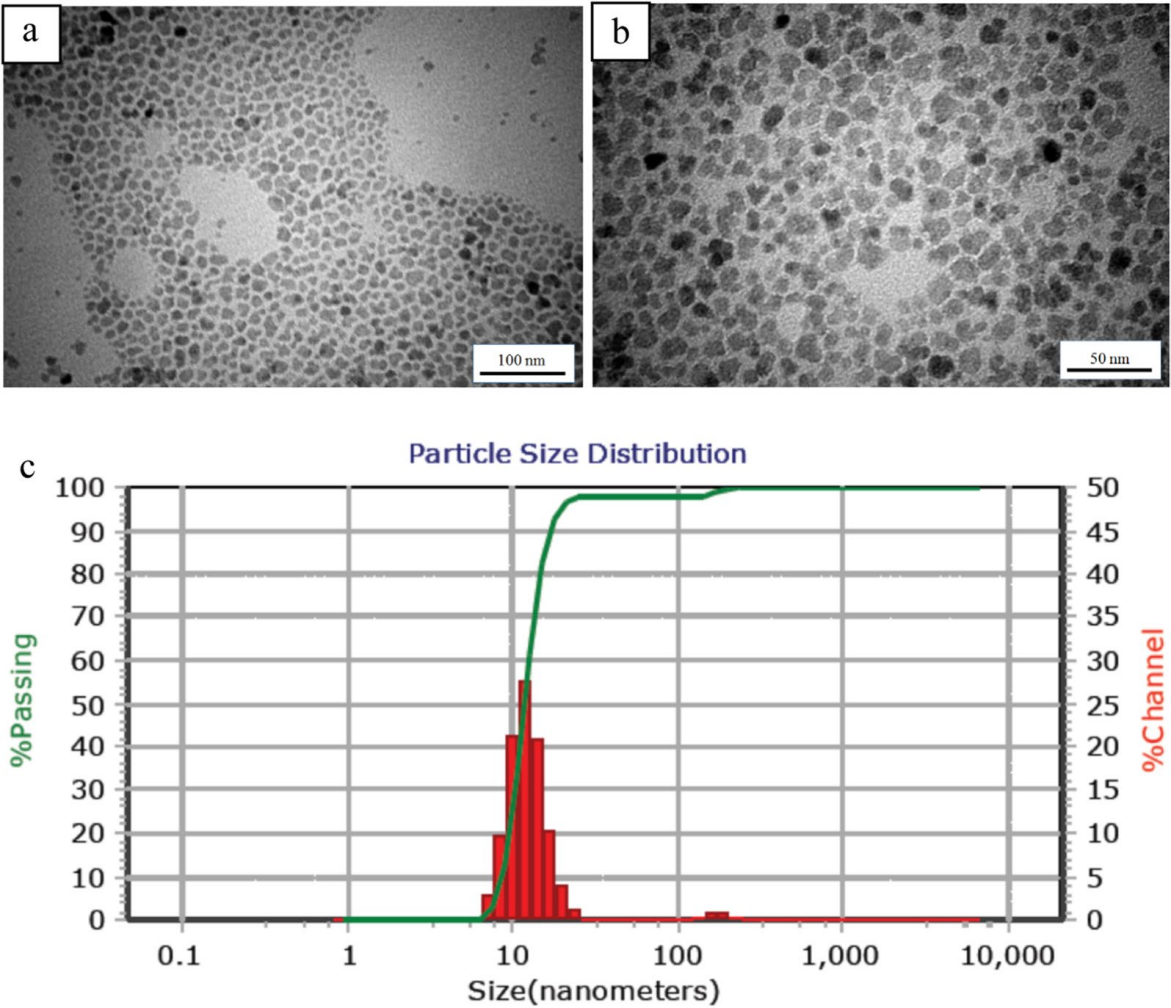
**a**, **b** TEM images of magnetic nanoparticles: **a** 100 nm, **b** 50 nm; **c** The size distribution and the average size of the modified IONPs

TEM images of oleic acid-coated magnetite nanoparticles are demonstrated in Fig. 2a, b. These images indicate that near-spherical nanoparticles with uniform size (approximately 10 nm) have been synthesized. It is also evident that the surface modification of nanoparticles with oleic acid did not meaningfully change the size of the nanoparticles. DLS analysis, which is based on the Brownian motion of the particles, provides quantitative results of the particle size and particle size distribution. Figure 2c illustrates a narrow size distribution in the range of 7.60–25.55 nm with an average of 11.8 nm, that is in good agreement with the TEM results.

**Fig. 3:**
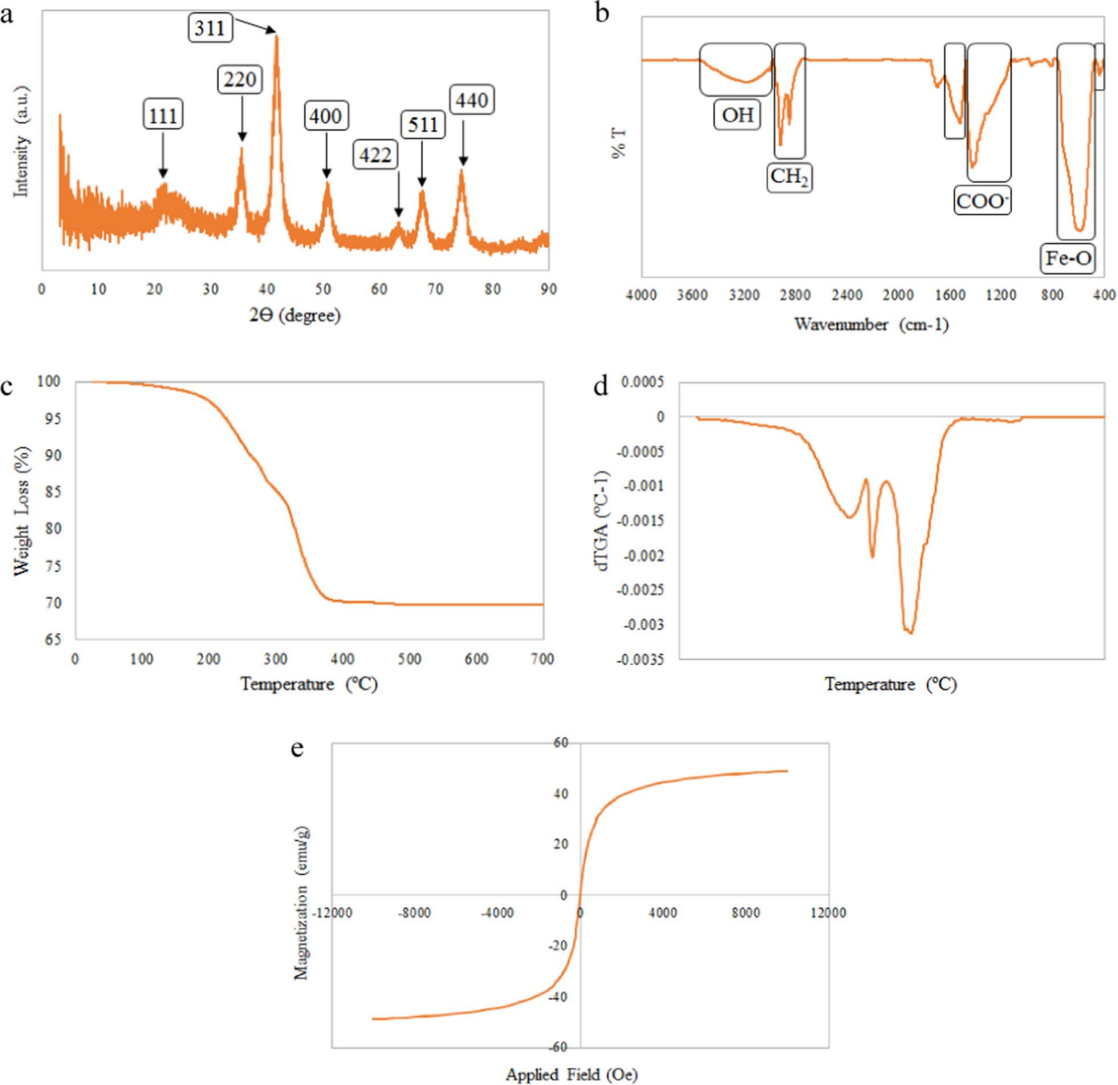
**a** XRD pattern; **b** FTIR spectrum; **c** TGA curve and **b** dTGA curve; **e** Magnetization properties of the IONPs

Figure 3a indicates the X-ray diffractogram of IONPs. The XRD pattern of the nanoparticles exhibits peaks at 2Ɵ = 21.6, 35.3, 41.6, 50.7, 63.3, 67.5, and 74.5° which are attributed to (111), (220), (311), (400), (422), (511), and (440) planes, respectively. The results are in accordance with the magnetite (JCPDS 19–629) reference patterns [46, 47].

Figure 3b shows the FTIR spectrum of the modified IONPs. The characteristic bands at 444 and 590 cm^−1^ are assigned to Fe–O bonding [48]. Two characteristic bands at 1430 and 1524 cm^−1^ (due to stretching vibration of COO^−^), confirm the presence of oleic acid on the nanoparticle surface [49]. Other characteristic absorption peaks at 2920 and 2850 cm^−1^ are related to the asymmetric and symmetric stretching of CH_2_ in oleic acid structure, respectively. The bending vibration of the OH band can be attributed to the absorption of water molecules on the surface of the IONPs [46, 49].

TGA analysis was employed to ensure the surface modification of the IONPs with oleic acid. Figure 3c, d demonstrates the TGA and the corresponding derivative (dTGA) curves of the coated IONPs. The weight loss observed at temperatures below 150 °C was negligible, which is assigned to the evaporation of the adsorbed water. The weight loss in the second transition region (temperature range 210–280 °C) has occurred owing to the elimination of the free or physically adsorbed oleic acid molecules [50]. The other weight loss observed at 333 °C is associated with the degradation of the oleic acid covalently bound to the IONP surface [51].

Figure 3e illustrates the field-dependent magnetization (M–H) curves of the IONPs. Results revealed the reversible field-dependent magnetization curves with no hysteresis loops, coercivity and remanent magnetization, which demonstrates that the net magnetization of the IONPs is zero in the absence of EMF [52]. These results express the super-paramagnetic behavior of the synthesized IONPs with high saturation magnetization value of 48.97 emu/g [46].

### Ag/IO nanocomposites evaluation

#### FE-SEM image

**Fig. 4:**
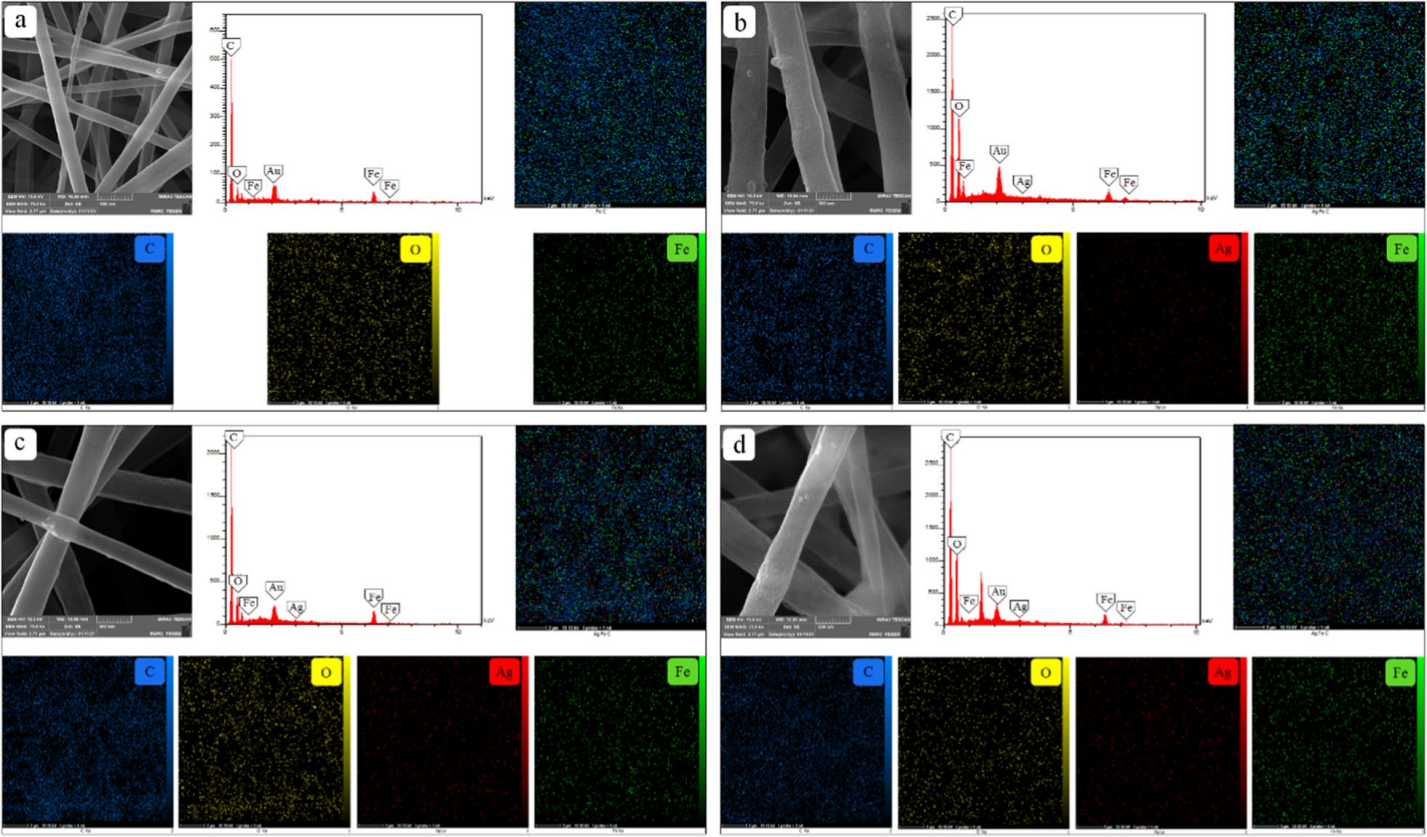
FE-SEM images, EDX, and element maps of nanocomposites synthesized at the fixed IONPs concentration (10%) and different concentrations of AgNPs; **a** 0, **b** 0.05, **c** 0.5, **d** 1%

**Fig. 5:**
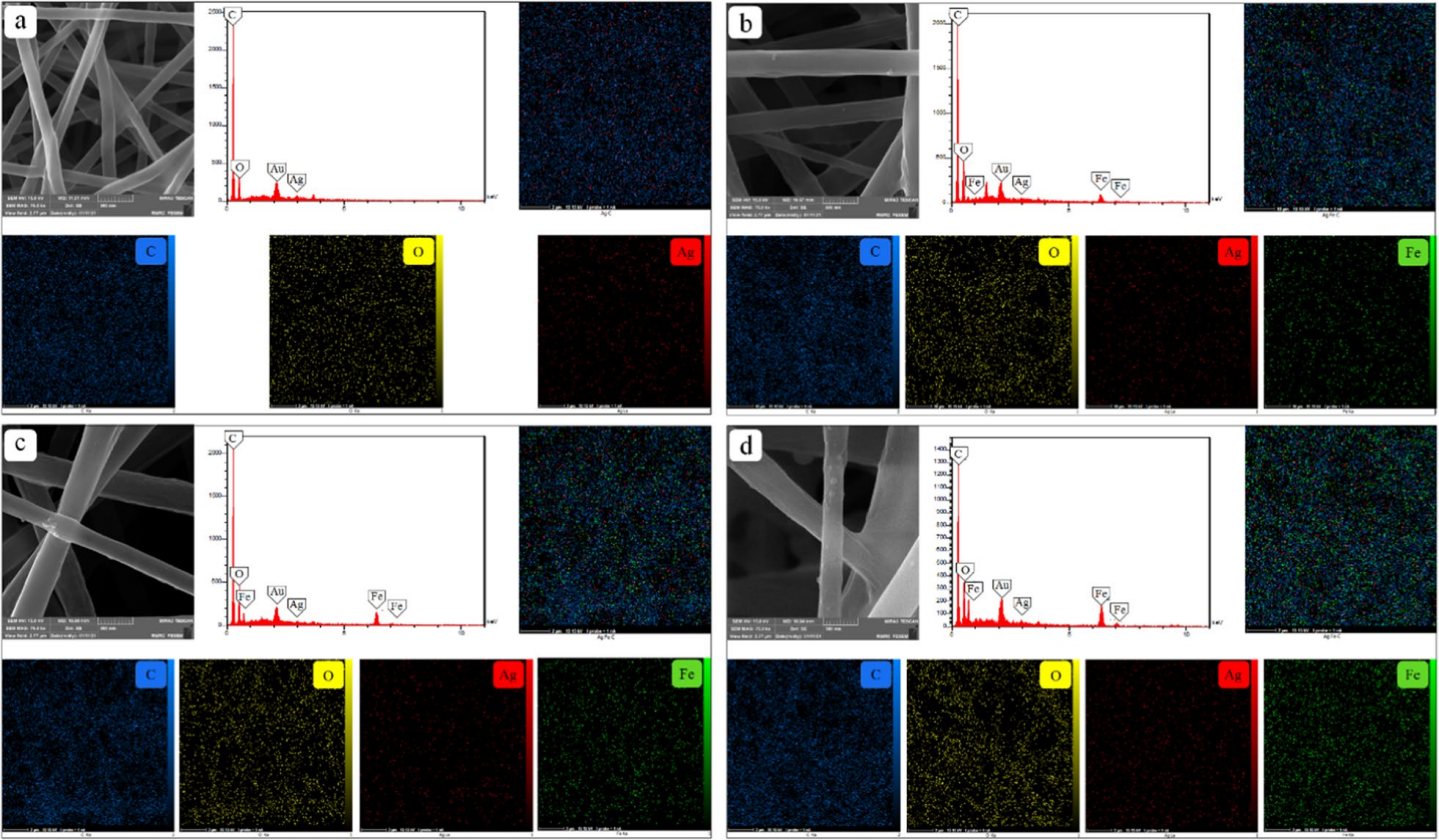
FE-SEM images, EDX, and element maps of the nanocomposites synthesized at the fixed AgNPs concentration (0.5%) and different concentrations of IONPs; **a** 0, **b** 5, **c** 10, **d** 15%

Figures 4 and 5 show the FE-SEM image, EDX, and the element maps of Ag and the IONPs in the synthesized nanocomposites. EDX analysis confirmed the presence of Ag and the IONPs throughout the nanocomposites. According to the FE-SEM and the element map images, spherical nanoparticles are distributed uniformly among the fibers without any agglomerations.

Figure 4a–d demonstrates the effect of four different concentrations of the AgNPs on the nanocomposites containing a fixed amount of the IONPs (10%). In Fig. 4a, C, O, and Fe elements can be identified in the maps. When silver nitrate was added to the composite, Ag also appeared in the results. Additionally, as shown in the element maps of Fig. 4a–d, higher concentrations of silver nitrate in the prepared solution leads to the increased content of AgNPs in the nanocomposite structure. The constant intensity of Fe peaks in EDX analysis, as well as the good distribution of this element in the maps (a-d), indicates uniform coating of the IONPs in samples 9, 10, 11, and 12. The same trend exists in Fig. 5a–d. The small diversity of Ag density in the element maps shows the fixed amount of AgNPs in samples 3, 7, 11, and 15 synthesized using a constant concentration of the silver nitrate in the precursor solution. In proportion to the IONPs concentration in the coating solution, the IONPs content in the composites increases.

#### Crystalline structure

**Fig. 6:**
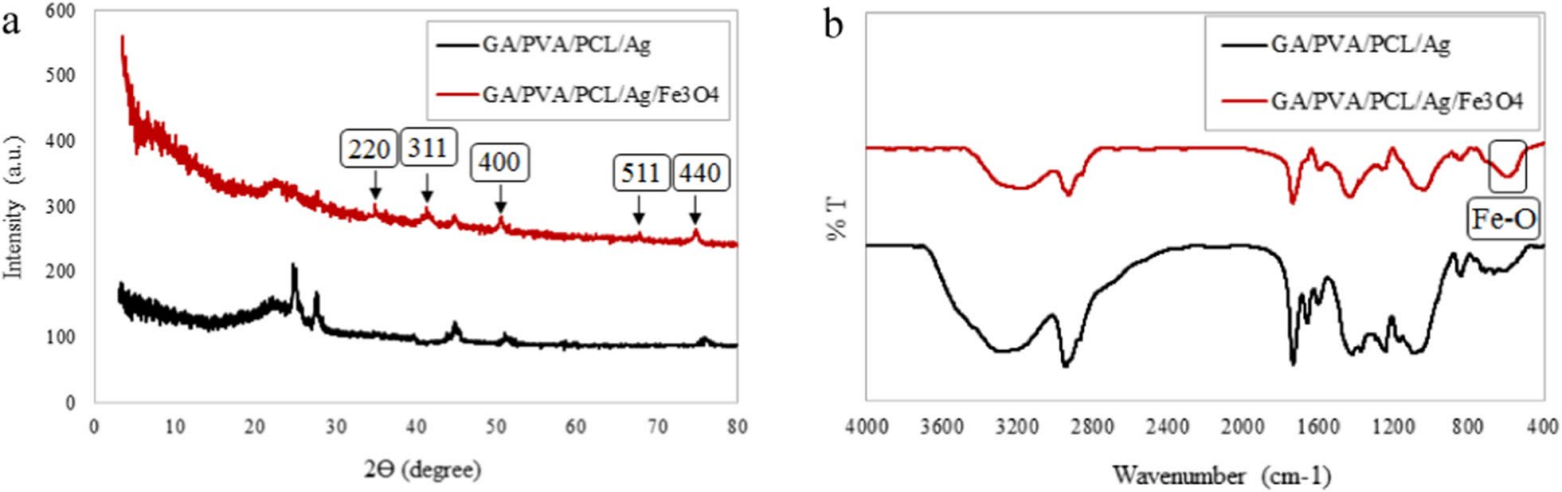
**a** XRD pattern, and **b** FTIR spectra of GA/PVA/PCL/Ag and GA/PVA/PCL/Ag/IO nanocomposites

Figure 6a demonstrates the X-ray diffractograms of GA/PVA/PCL/Ag and GA/PVA/PCL/Ag/Fe_3_O_4_ nanocomposites. The addition of the IONPs to the nanocomposites has reduced the intensity of the peaks at 2θ = 24.95 & 27.69°, due to the interaction between PCL and the IONPs [53]. The IONP-containing nanocomposites showed three new peaks at 2θ = 34.82, 41.51 and 67.69° (for (220), (311), and (511) planes). Moreover, the intensity of the peaks increases at 2θ = 50.53 & 74.79° due to (400) and (440) planes, respectively.

#### Chemical structure

FTIR spectra of GA/PVA/PCL/Ag and GA/PVA/PCL/Ag/Fe_3_O_4_ nanocomposites are shown in Fig. 6b. The main difference is the peak at 597 cm^−1^. This peak is related to the stretching vibration of the metal–oxygen absorption band (Fe–O bond), indicative of the presence of the magnetite nanoparticles. Additionally, interactions between the IONPs and the nanocomposites can change the intensities or the peak shift. In the presence of the IONPs, the peaks of the hydroxyl group, C–H (asymmetric and stretching), C=O stretching vibration, COO^−^ symmetric stretching, and C–C stretching vibrations have been shifted to 3187, 2927, 1733, 1433, and 846 cm^−1^, respectively [54].

#### Magnetic properties

**Fig. 7:**
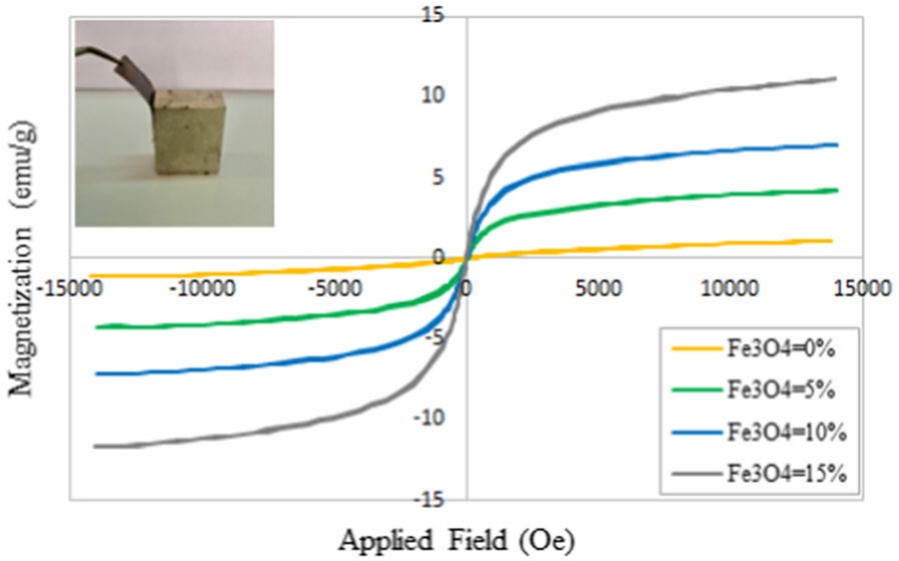
The magnetization properties of the IONPs-containing nanocomposites

The magnetization–magnetic field (M–H) curves of the nanocomposites at 300 K are shown in Fig. 7. The zero coercivity and the reversible hysteresis behavior of the nanocomposites reveal their super-paramagnetic property, which occurs only at the nanoscale. The saturation magnetization value of the IONPs was 48.97 emu/g, which was reduced to 4.23, 6.98, and 11.16 emu/g for the nanocomposites containing three different concentrations of the IONPs (5, 10, and 15%, respectively). However, the result also exhibits super-paramagnetic property [52, 55, 56]. Results demonstrate higher saturation magnetization values compared to the other researchers' studies such as Ahn and Kang [57]. Using the coating method instead of merging the nanoparticles into the solution prepared for the electrospinning process, increases the particle size and improves the magnetic property since magnetic characterization depends on the size of the synthesized nanoparticles [58].

#### Antibacterial activity

The effects of AgNPs content, IONPs concentration, and applying EMF, on the antibacterial activity of the nanocomposites against different microbial strains (*S. aureus* and *methicillin resistant Staphylococcus aureus (MRSA)* (Gram-positive), *P. aeruginosa* and *E. coli* (Gram-negative), and *C. albicans* (Yeast)), were investigated using two methods of disk diffusion and colony counting. Due to a large number of samples, only 5 samples include sample 1 (control sample), sample 4 (containing the highest amount of silver nanoparticles), sample 13 (containing the highest amount of iron oxide nanoparticles), sample 6 (containing the lowest amount of both silver nanoparticles and iron oxide) and Sample 16 (containing the highest amount of both silver nanoparticles and iron oxide) were used for microbial strains of *MRSA*, *E. coli*, and *C. albicans*.

**Fig. 8:**
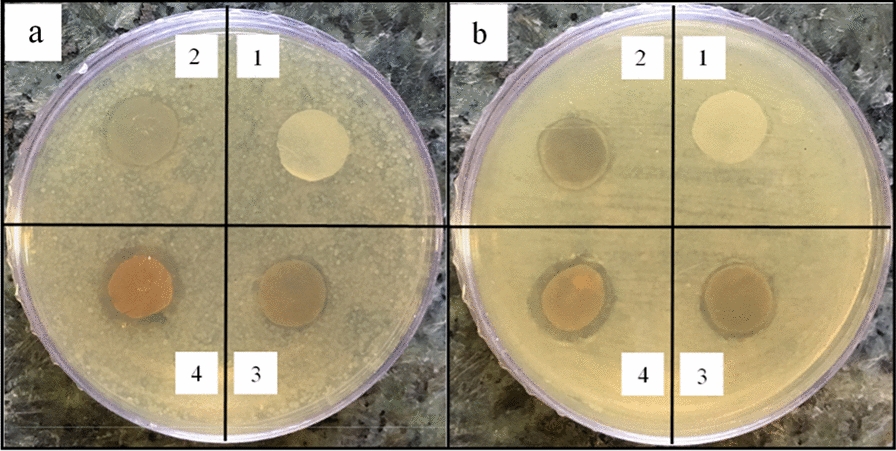
Antibacterial activity of IONPs-free nanocomposites containing 0, 0.05, 0.5 and 1% AgNPs (samples 1, 2, 3 and 4, respectively) against **a** *Staphylococcus aureus*, and **b** *Pseudomonas aeruginosa*

**Fig. 9:**
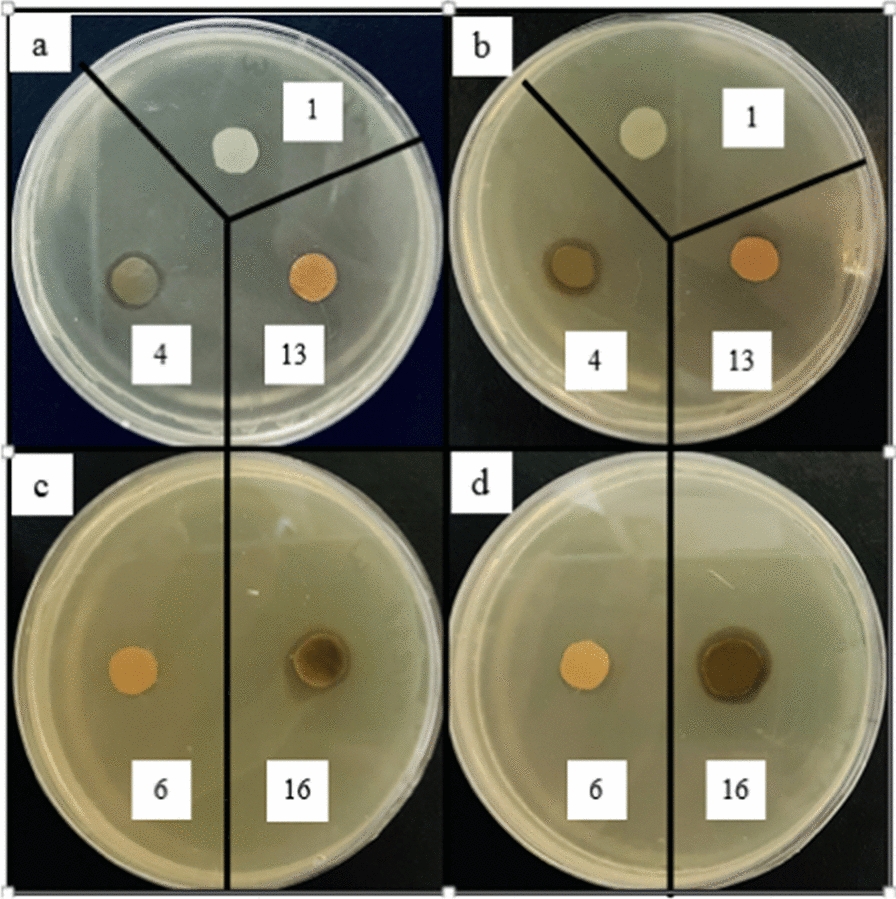
Antibacterial activity of Ag/IO nanocomposites against *MRSA*, **a** and **c** in the absence and **b** and** d** in the presence of EMF

**Fig. 10:**
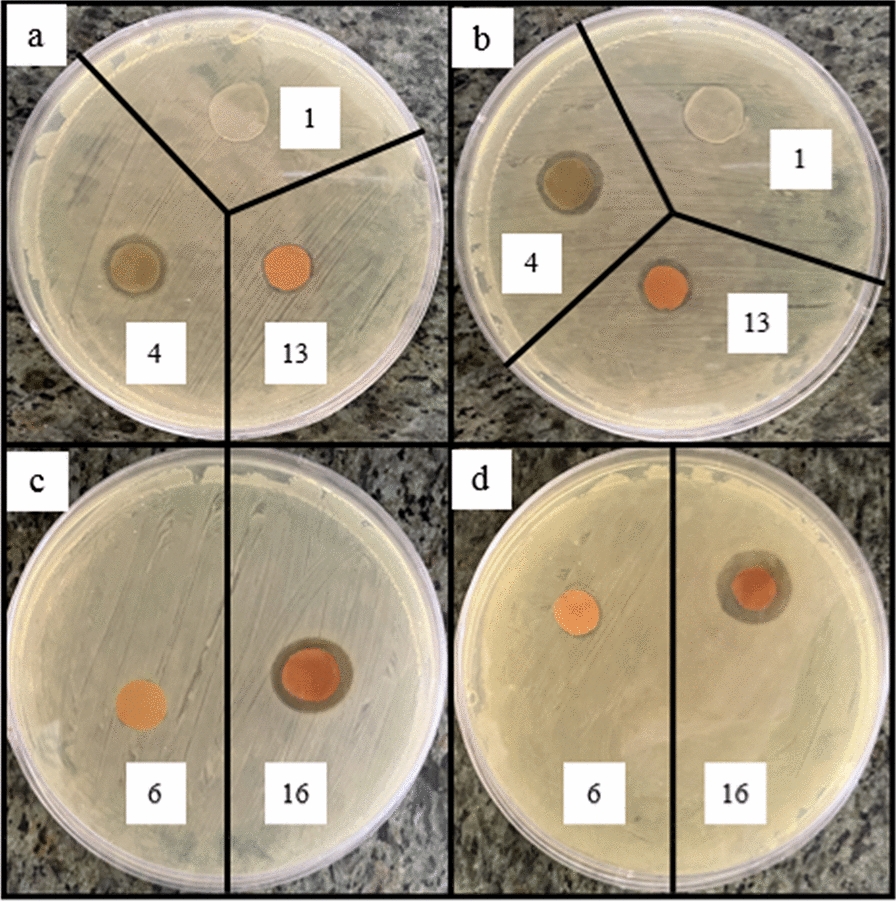
Antibacterial activity of Ag/IO nanocomposites against *E.Coli*, **a** and **c** in the absence and **b** and **d** in the presence of EMF

**Fig. 11:**
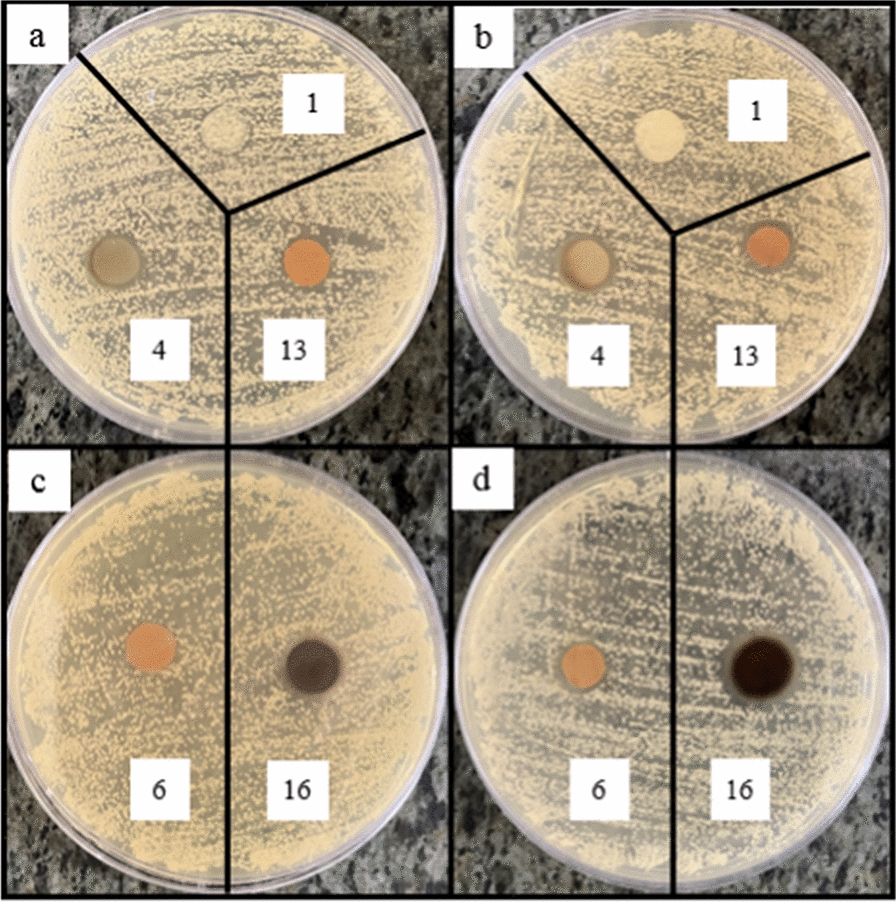
Antibacterial activity of Ag/IO nanocomposites against *C. Albicans*, **a** and **c** in the absence and **b** and **d** in the presence of EMF

According to Fig. 8a, b, the nanocomposites containing 0.05% AgNPs show low antibacterial activity. By increasing the concentration of AgNPs (to 1%), the diameter of the inhibition zone increases since more AgNPs can be released and higher antibacterial activity can be obtained (Figs. 9a, b, 10a, b, 11a, b) [59, 60]. AgNPs adhesion to the cell wall through electrostatic interactions causes its damage. In the following, it penetrates the cell and pushes the cell content out, which results in DNA deformation and disrupting its transcription and translation, and the generation of reactive oxygen species (ROS) and free radicals [13, 61].

**Fig. 12:**
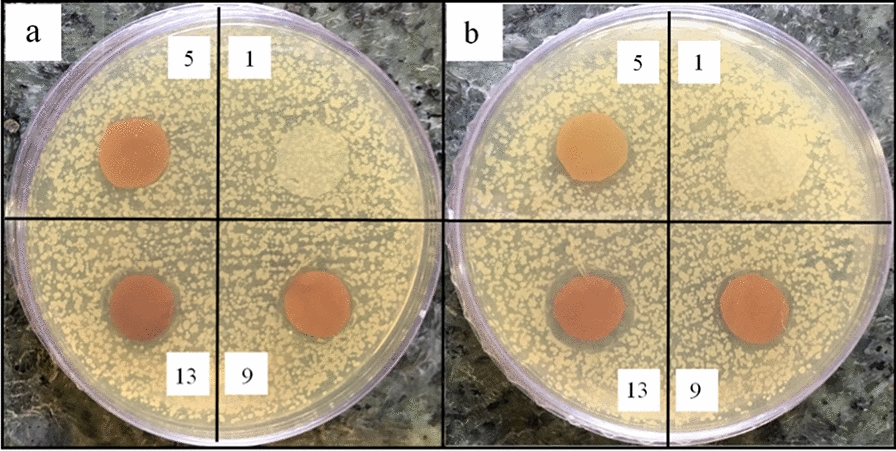
Antibacterial test results of AgNPs-free nanocomposites containing 0, 5, 10 and 15% IONPs (samples 1, 5, 9, and 13, respectively) against *Staphylococcus aureus*, **a** in the absence and **b** in the presence of EMF

The content of IONPs in the nanocomposite is another influential factor affecting antibacterial activity. Figures 9a, b, 10a, b, 11a, b, 12a, b shows that increasing the IONPs content from 5 to 15% leads to increased antibacterial activity. The main mechanism of the antibacterial activity of IONPs is the oxidative stress caused by ROS. ROS involve superoxide radicals (O_2_^−^), hydroxyl radicals (–OH), singlet oxygen (^1^O_2_), and hydrogen peroxide (H_2_O_2_), which penetrate the bacteria cells and damage their proteins and DNA [62–64]. On the other hand, bacteria use adhesive surface structures to attach to tissues. The attachment of IONPs to the cell wall and surface structures of bacteria causes the bacterial adherence factors to be occupied and inactivated, preventing them from binding [65].

**Fig. 13:**
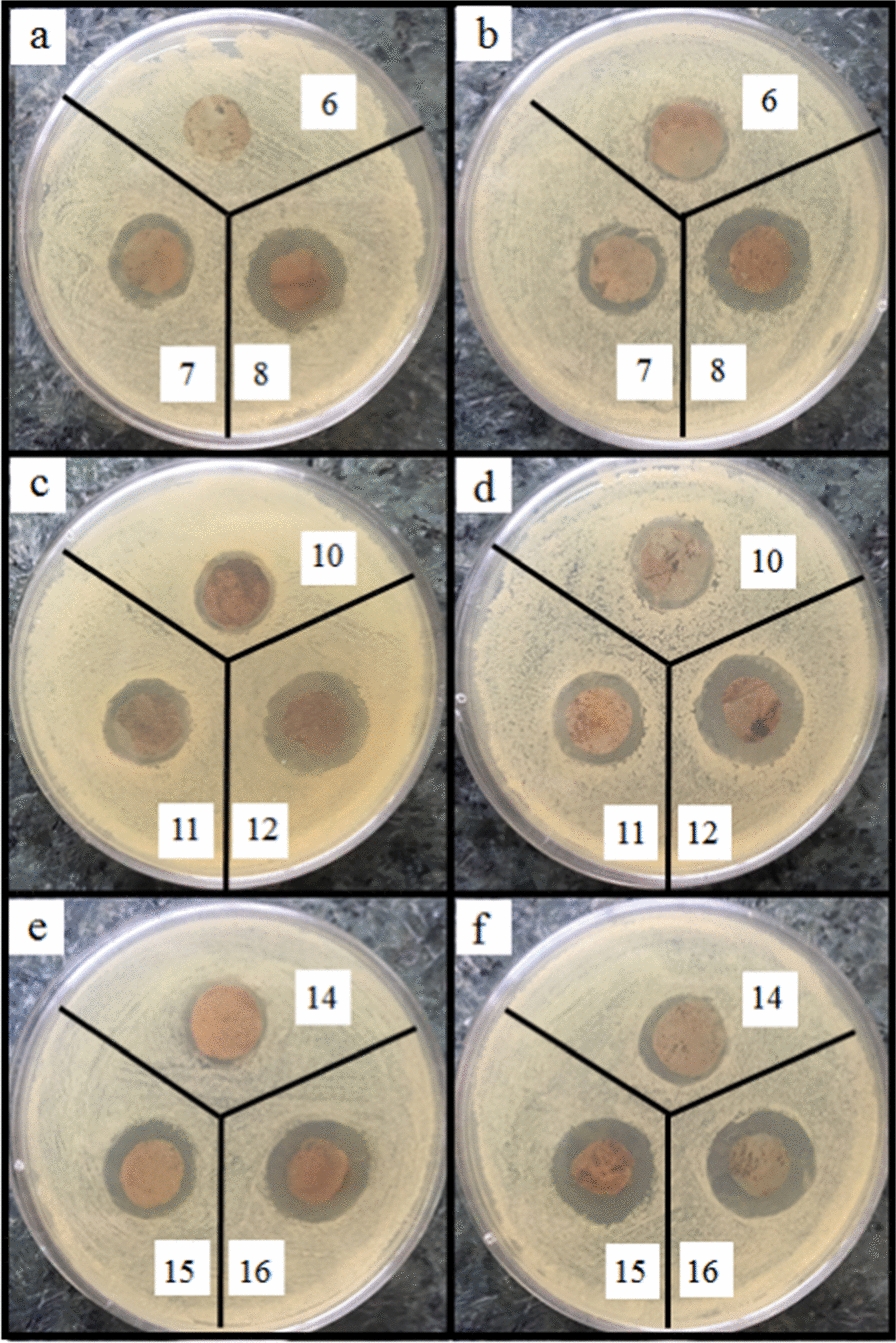
Antibacterial activity of Ag/IO nanocomposites containing 0.05, 0.5, and 1% AgNPs; and 5% (**a** and **b**), 10% (**c** and **d**), and 15% IONPs (**e** and **f**) against *S. aureus*, (**a**, **c** and **e**) in the absence and (**b**, **d**, and **f**) in the presence of EMF

**Fig. 14:**
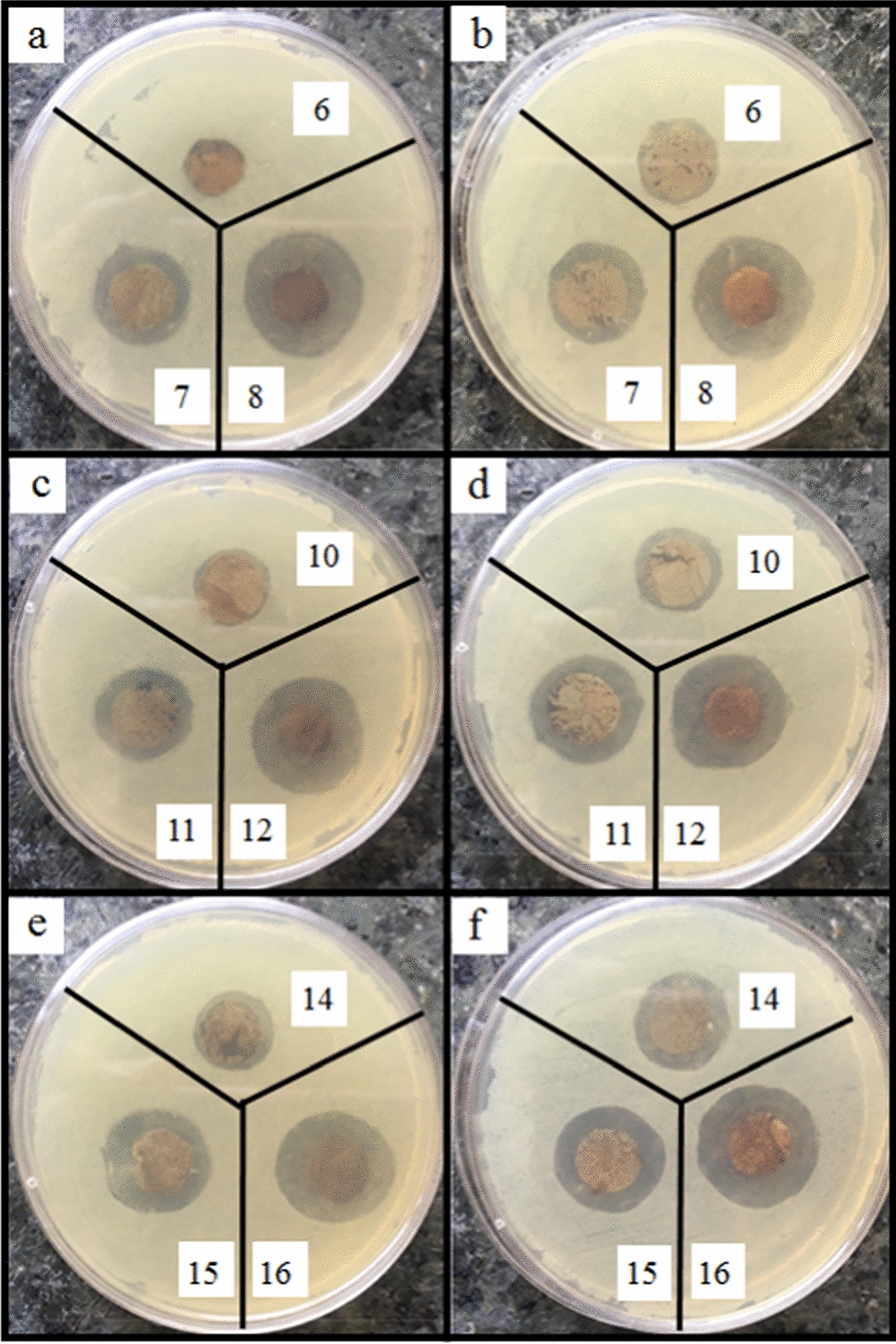
Antibacterial activity of Ag/IO nanocomposites containing 0.05, 0.5, and 1% AgNPs; and 5% (**a** and **b**), 10% (**c** and **d**), and 15% IONPs (**e** and **f**) against *P. aeruginosa*, (**a**, **c** and **e**) in the absence and (**b**, **d**, and **f**) in the presence of EMF

A comparison of the images in Figs. 9c, d, 10c, d, 11c, d, 13, 14 and the data in Tables 1, 2, 3, 4 and 5 demonstrates that the presence of EMF increases the antibacterial activity of IONPs-containing nanocomposites. The magnetic moments of the IONPs are randomly oriented in the absence of EMF. Therefore, net magnetization is zero. Applying a strong enough EMF forces the magnetic moments of the IONPs to align along the magnetic field direction [66].

**Table 1:** Inhibition zone diameter (mm) for *S. aureus*

IONPs concentration (%)	AgNPs concentration (%)		
	0.05	0.5	1
5			
A EMF^*^	10 ± 0.06	11.8 ± 0.15	13 ± 0.06
P EMF^**^	11 ± 0.1	12.8 ± 0.06	14.1 ± 0.1
10			
A EMF^*^	11.5 ± 0.1	12 ± 0.12	13.5 ± 0.1
P EMF^**^	12.5 ± 0.15	13 ± 0.06	14.5 ± 0.1
15			
A EMF^*^	12 ± 0.15	12.9 ± 0.1	14 ± 0.06
P EMF^**^	12.5 ± 0.1	13.8 ± 0.1	15 ± 0.15

A EMF *: absence of external magnetic field

P EMF **: presence of external magnetic field

**Table 2:** Inhibition zone diameter (mm) for *P. aeruginosa*

IONPs concentration (%)	AgNPs concentration (%)		
	0.05	0.5	1
5			
A EMF^*^	10 ± 0.06	12.5 ± 0.15	15 ± 0.15
P EMF^**^	11.2 ± 0.21	13.1 ± 0.15	16 ± 0.06
10			
A EMF^*^	10.5 ± 0.1	13 ± 0.06	15.5 ± 0.1
P EMF^**^	11.6 ± 0.06	14.2 ± 0.1	16.5 ± 0.06
15			
A EMF^*^	11 ± 0.15	13.7 ± 0.12	16 ± 0.1
P EMF^**^	12.4 ± 0.06	15.1 ± 0.15	16.8 ± 0.12

A EMF *: absence of external magnetic field

P EMF **: presence of external magnetic field

**Table 3:** Inhibition zone diameter (mm) for *MRSA*

IONPs concentration (%)	AgNPs concentration (%)			
	0	0.05	0.5	1
0	0	*–*	*–*	12.8 ± 0.06
5				
A EMF^*^	–	10 ± 0.06	–	–
P EMF^**^	–	10.8 ± 0.1	–	–
10				
A EMF^*^	–	–	–	–
P EMF^**^	–	–	–	–
15				
A EMF^*^	11 ± 0.15	–	–	14 ± 0.06
P EMF^**^	12 ± 0.1	–	–	14.9 ± 0.15

A EMF *: absence of external magnetic field

P EMF **: presence of external magnetic field

**Table 4:** Inhibition zone diameter (mm) for *E. Coli*

IONPs concentration (%)	AgNPs concentration (%)			
	0	0.05	0.5	1
0	0	*–*	*–*	13.6 ± 0.1
5				
A EMF^*^	–	10 ± 0.06	–	–
P EMF^**^	–	11 ± 0.15	–	–
10				
A EMF^*^	–	–	–	–
P EMF^**^	–	–	–	–
15				
A EMF^*^	12 ± 0.15	–	–	15 ± 0.06
P EMF^**^	13 ± 0.06	–	–	15.8 ± 0.1

A EMF *: absence of external magnetic field

P EMF **: presence of external magnetic field

**Table 5:** Inhibition zone diameter (mm) for *C. Albicans*

IONPs concentration (%)	AgNPs concentration (%)			
	0	0.05	0.5	1
0	0	*–*	*–*	13.2 ± 0.06
5				
A EMF^*^	–	10 ± 0.06	–	–
P EMF^**^	–	11 ± 0.1	–	–
10				
A EMF^*^	–	–	–	–
P EMF^**^	–	–	–	–
15				
A EMF^*^	12 ± 0.15	–	–	14.2 ± 0.15
P EMF^**^	13 ± 0.1	–	–	15 ± 0.15

A EMF *: absence of external magnetic field

P EMF **: presence of external magnetic field

Due to the reasonable amount of the IONPs in the nanocomposites and their superparamagnetic properties, the applied EMF increases the IONPs release and improves the antibacterial activity. Moreover, the controlled release of IONPs leads to the more accessible release of the AgNPs, due to the increased porosity, and thus the better antibacterial activity of the nanocomposites [67, 68].

In addition to the disc diffusion assay, the antibacterial activity of the nanocomposites against two bacterial strains of *S. aureus* and *P. aeruginosa* was also carried out through the colony counting method, which is shown in Table 6.

**Table 6:** Bacterial Growth Inhibition (%) of the nanocomposites against *S. aureus*, and *P. aeruginosa*

	Bacterial growth inhibition (%)				
Bacteria	4		6	13	16
	A EMF				
*S. aureus*	98.82	89.23		94.62	99.91
*P. aeruginosa*	98.92	90.00		95.74	99.92
	P EMF				
*S. aureus*	98.82	97.31		98.65	99.98
*P. aeruginosa*	98.92	98.33		98.83	99.99

A EMF *: absence of external magnetic field

P EMF **: presence of external magnetic field

From this table it is obvious that the number of bacterial colonies of petri dishes corresponding to nanocomposites containing silver and iron oxide nanoparticles for both pathogenic bacteria is significantly lower than the control nanocomposite (sample 1). The results show that the addition of silver nanoparticles (sample 4), the addition of iron oxide nanoparticles (sample 13), the simultaneous integration of both nanoparticles (samples 6 and 13), and the use of a magnetic field reduce the number of bacterial colonies. These results are in agreement with agar disk diffusion analysis.

Among the microbial strains, *P.aeruginosa* and *MRSA* showed the highest and lowest sensitivity to the antibacterial nanocomposites, respectively. The observed differences can be attributed to the presence of a thick layer of peptidoglycan in the cell wall of gram-positive bacteria, which acts like a barrier against the penetration of the antibacterial nanoparticles into the bacteria and affects their performance [39, 69].

#### ROS generaion

**Fig. 15:**
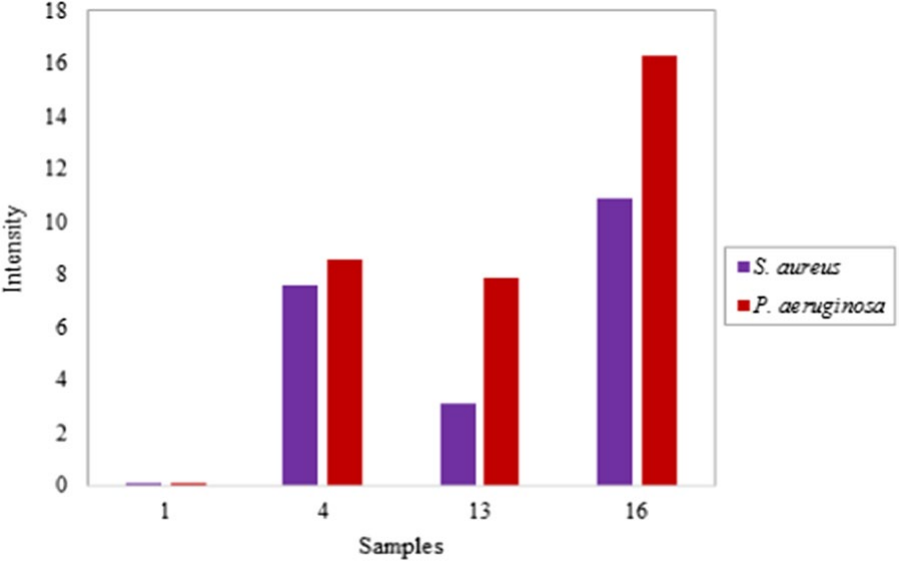
Effect of Ag/IO NPs-containing nanocomposites on ROS formation in presence of *S. aureus* and *P. aeruginosa* bacterial cells

ROS generation was measured by the DCFH-DA assay after exposing the nanocomposites to *S. aureus* and *P. aeruginosa* to ascertain whether it has an effect on the antibacterial mechanism of Ag/IO nanocomposites or not. The results revealed that NPs-containing nanocomposites, produced more ROS compared to untreated nanocomposite, in the presence of both *S. aureus* and *P. aeruginosa* in a concentration-dependent manner. Increasing the concentration of AgNPs to 1% leads to about 8% increase in ROS as compared to NPs-free nanocomposites. Similarly, bacteria exposed to IONPs-containing nanocomposites demonstrated increased ROS production (Fig. 15).

Altogether, the above data suggest that AgNPs act as oxidative stress inducer agents and cause the loss of cell viability in *S. aureus* and *P. aeruginosa* bacteria through ROS generation. ROS formation of AgNPs-containing nanocomposites can be assigned to the higher silver ion release. The interaction between metal NPs and bacterial cells often leads to ROS production, which damage to proteins and nucleic acids. The IONPs also produce ROS at their surface and increasing the concentration, causes increased ROS formation as more NPs are released from the nanocomposites [39, 70]. Taken together, the above finding suggest that ROS formation is a possible mechanism responsible for bacterial cell death in presence of Ag/IO nanocomposites.

#### Cytotoxicity

**Fig. 16:**
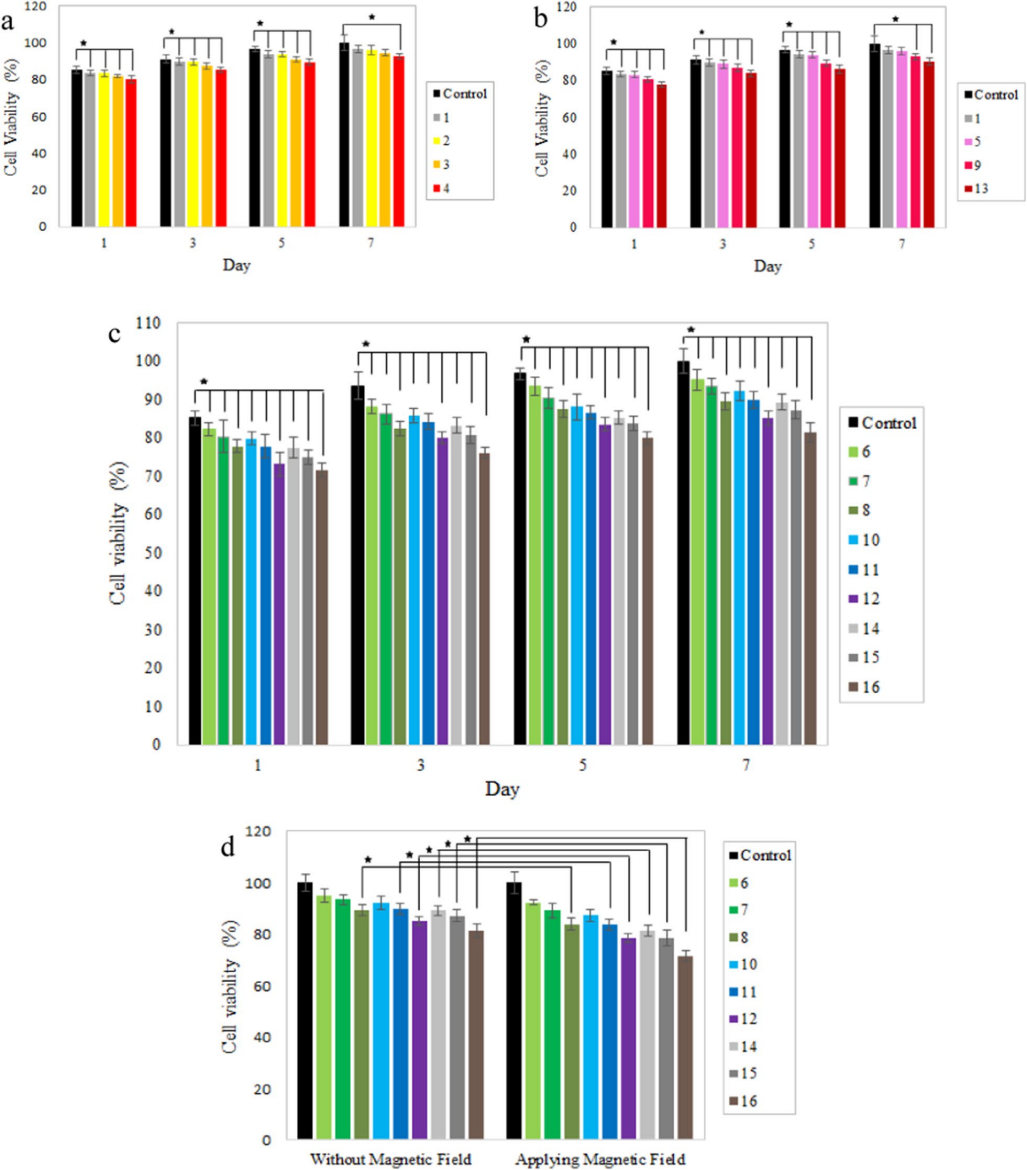
MTT assay results of the **a** IONPs-free nanocomposites containing 0, 0.05, 0.5, and 1% AgNPs; **b** AgNPs-free nanocomposites containing 0, 5, 10, and 15% IONPs; **c** Ag/IO nanocomposites containing 0.05, 0.5, and 1% AgNPs; and 5, 10, and 15% IONPs on days 1, 3, 5 and 7; **d** Ag/IO nanocomposites containing 0.05, 0.5, and 1% AgNPs; and 5, 10, and 15% IONPs on the 7th day in the absence and presence of EMF. Data are reported as mean ± SD (n = 5 and *p < 0.05)

**Fig. 17:**
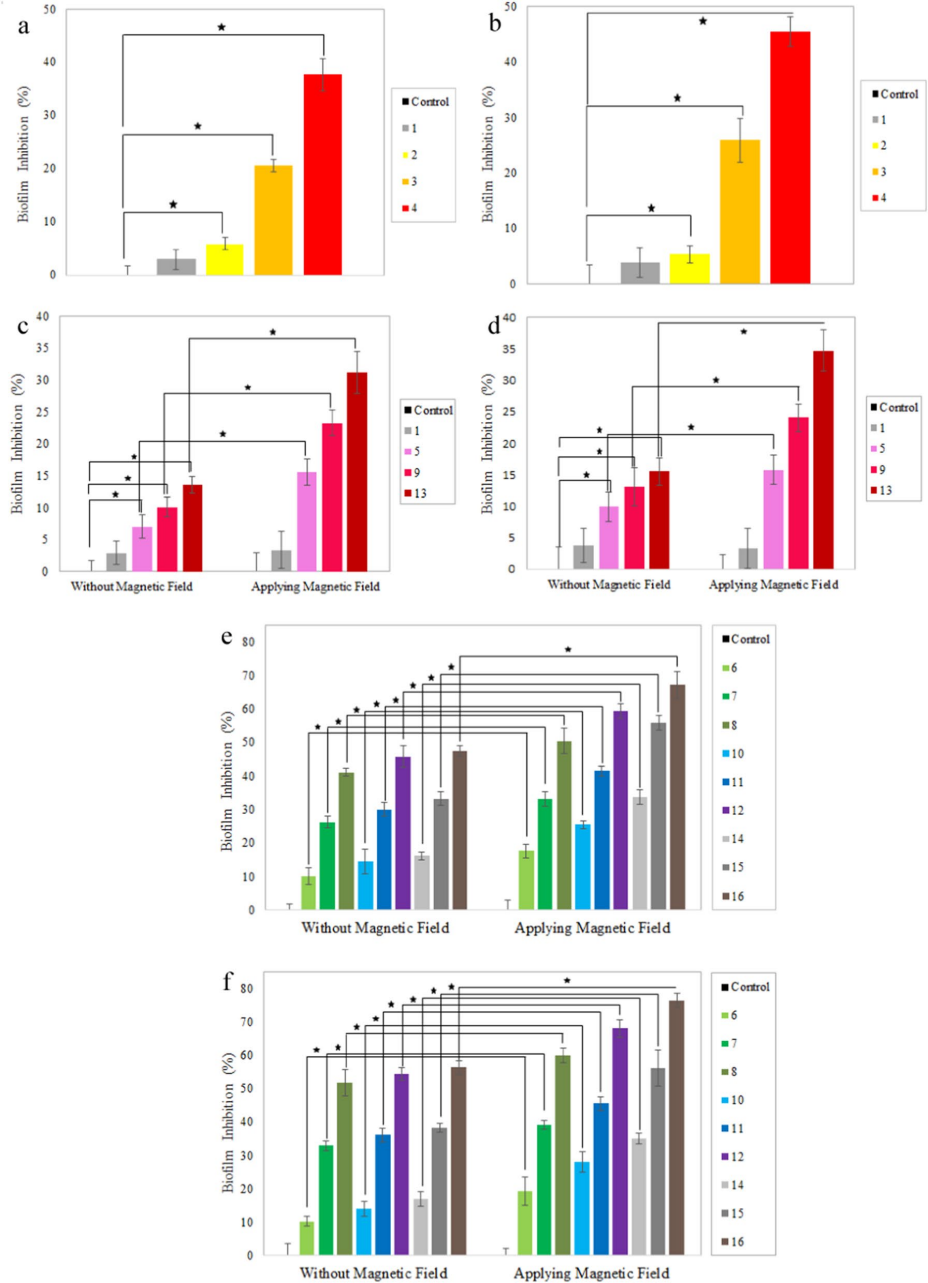
Cell viability (%) of macrophage cells at different time incubation

Cytotoxicity assay was carried out to study the biocompatibility of AgNPs, IONPs, and EMF on the fibroblast and macrophage cells. Cells were cultured on the nanocomposites for 1, 3, 5, and 7 days to perform the MTT assay. Generally, the type of nanoparticles, their shape, size, dose, surface properties, and the ways they are added to the system affect their toxicity [58]. According to Figs. 16a, b and 17, cells were able to grow on all nanocomposites; but increasing AgNPs and IONPs contents in the nanocomposites reduced cell proliferation due to the more released silver and iron oxide nanoparticles. Results revealed dose-dependent cytotoxicity of the silver nanoparticles [4, 16]. Increasing the AgNPs concentration to 1% reduced cell viability of fibroblasts and macrophages to about 80%. Ankamwar et al. showed that IONPs are not toxic in the concentration range of 0.1–10 µg/mL^−1^, but cell viability decreases when the concentration reaches 100 µg/mL^−1^ [71] and our study is in agreement with the mentioned report. Increasing the concentration of IONPs from 5 to 15% on the first day, caused a decrease in fibroblast viability from 83.1 to 77.8%. A low concentration of IONPs caused no toxicity in macrophages, even slight growth promotion is observed in prior research [72]. At the concentration of 15% IONPs phagocytic cells exhibited a higher survival rate of 85.1% [73]. According to previous reports, higher concentrations of IONPs could exert cytotoxicity by the generation of excessive reactive oxygen species (ROS). Additionally, IONPs impair cellular activities and cell growth by adsorbing proteins and other nutrients of the cell culture medium [72, 74].

Figures 16c and 17 shows the simultaneous effect of AgNPs and IONPs on the biocompatibility of the nanocomposites. As presented in these figure, when both NPs are integrated into the nanocomposite, their cytotoxicity increases. Moreover, the nanocomposites containing high concentrations of AgNPs and IONPs on days 1 and 3 showed higher levels of toxicity, and the cell viability reduced to less than 80% for both cell types. However, cell viability was improved even for the nanocomposites containing the maximum amounts of the nanoparticles (sample 16) because of the considerable increase in the cell proliferation from day 5. Therefore, the incorporation of AgNPs and IONPs does not considerably affect the biocompatibility of the nanocomposites.

According to Fig. 16d, applying an EMF to the nanocomposites with a low concentration of the nanoparticles has no considerable effect on the cell viability, but the nanocomposites containing 15% IONPs release more IONPs and AgNPs and consequently increase the system's cytotoxicity [75].

Generally, due to the larger size of eukaryotic cells than prokaryotes and their structural differences, higher concentrations of nanoparticles are required to cause cytotoxicity in them. Using nanoparticles in low concentrations that are effective against microorganisms, has no toxic effect on the eukaryotic cells [76].

#### Antibiofilm activity

More than 60% of bacterial infections are caused by the formation of biofilms in chronic wounds. Unlike planktonic bacteria, which are easily killed, biofilms attach to the surface and are resistant to multiple antibiotics, which makes their inhibition difficult. Therefore, in this study, the inhibitory effect of Ag/IO nanocomposites against *S.aureus* and *P.aeruginosa* biofilms was investigated through a crystal violet staining assay.

**Fig. 18:**
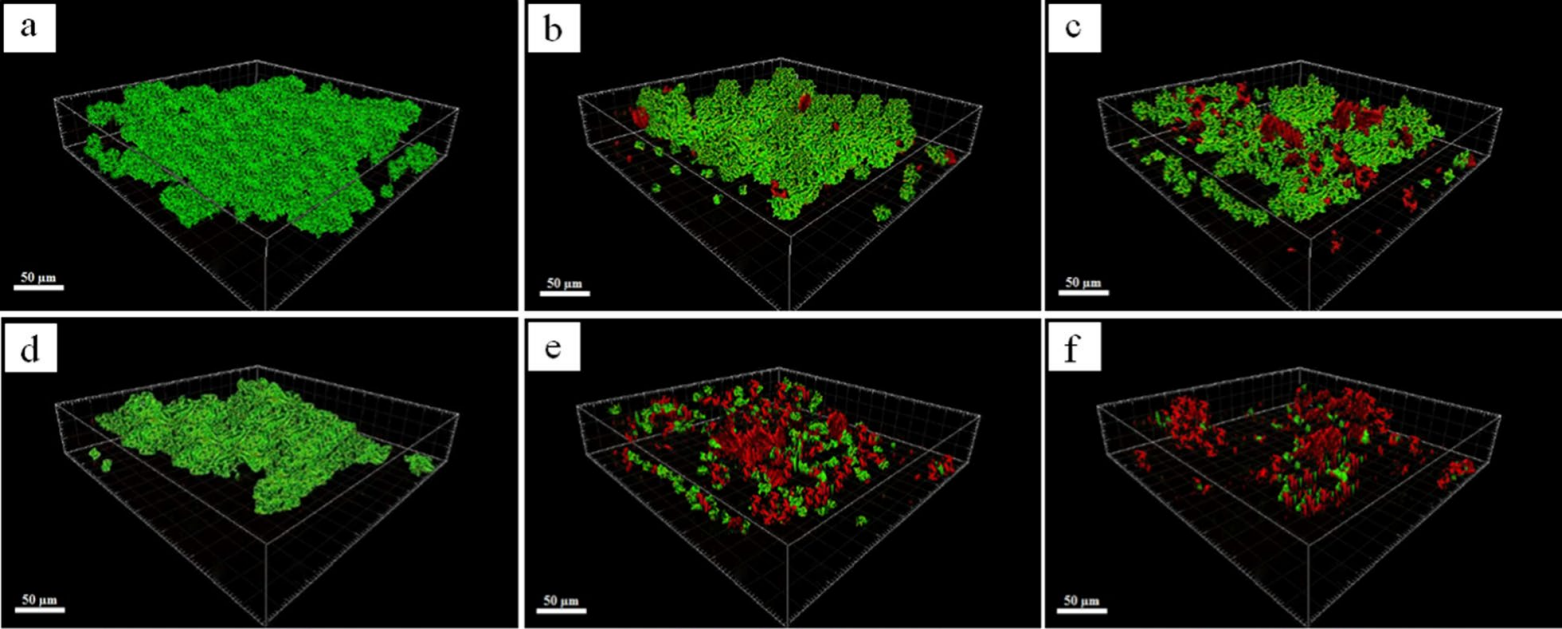
Antibiofilm analysis of **a**, **b** IONPs-free nanocomposites containing 0, 0.05, 0.5, and 1% AgNPs (samples 1, 2, 3, and 4) against **a** *S. aureus*, and **b** *P. aeruginosa*; **c**, **d** AgNPs-free nanocomposites containing 0, 5, 10, and 15% IONPs (samples 1, 5, 9 and 13) in the absence and presence of EMF against **c** *S. aureus*, and **d** *P. aeruginosa*; **e**, **f** Ag/IO nanocomposites containing 0.05, 0.5, and 1% AgNPs; and 5, 10, and 15% IONPs in the absence and presence EMF against **e** *S. aureus*, and **f** *P. aeruginosa*. Data are reported as the mean ± SD (n = 5 and *p < 0.05)

According to Fig. 18a, b, the nanoparticles-free nanocomposites (sample 1) have no significant effect on the biofilm inhibition, but the addition of small amounts of AgNPs (0.05%) to the nanocomposites, resulted in 6 and 5% biofilm inhibition by *S.aureus* and* P.aeruginosa*, respectively; while AgNPs content increases up to 1%, promotes the biofilm inhibition to 38 and 45%, respectively [16, 77, 78].

Figure 18c, d shows the effect of different concentrations of IONPs on biofilm inhibition. The nanocomposites containing 5, 10, and 15% IONPs inhibit 7, 10, and 14% of *S. aureus* biofilm, respectively. The same trend was observed for *P. aeruginosa*, and with an increase in IONPs content from 5 to 10% and 15%, biofilm inhibition boosts from 10 to 13% and 15%, respectively [79]. There are several mechanisms of biofilm inhibition and antibacterial activity of IONPs, one of which is the generation of ROS. In addition, the electrostatic interactions between the nanoparticles and the bacterial cell wall leading to its destruction and bacterial death [62].

As presented in Fig. 18e, f, the biofilm inhibition increments with increasing silver and iron oxide nanoparticles. A comparison of the results shows that an increase of 0.5% in the amount of AgNPs has a more remarkable effect than an increase of 5% in the amount of IONPs.

A comparison of the nanocomposites manifests that applying EMF significantly enhances the inhibitory effect due to the increased release of IONPs and consequently AgNPs. As a result, more AgNPs penetrate the biofilm, leading to further inhibition. In addition to the antibacterial properties, IONPs in the presence of EMF can mechanically destroy the biofilm structure by penetrating it and showing more inhibitory effects. Additionally, in the presence of EMF, iron oxide nanoparticles can be more permeable into the bacterial cell wall by converting the magnetic energy into heat [80]. The highest inhibitory activity of the nanocomposites against the biofilm of *S. aureus* and *P. aeruginosa* was in sample 16, increased significantly upon applying EMF.

**Fig. 19:**
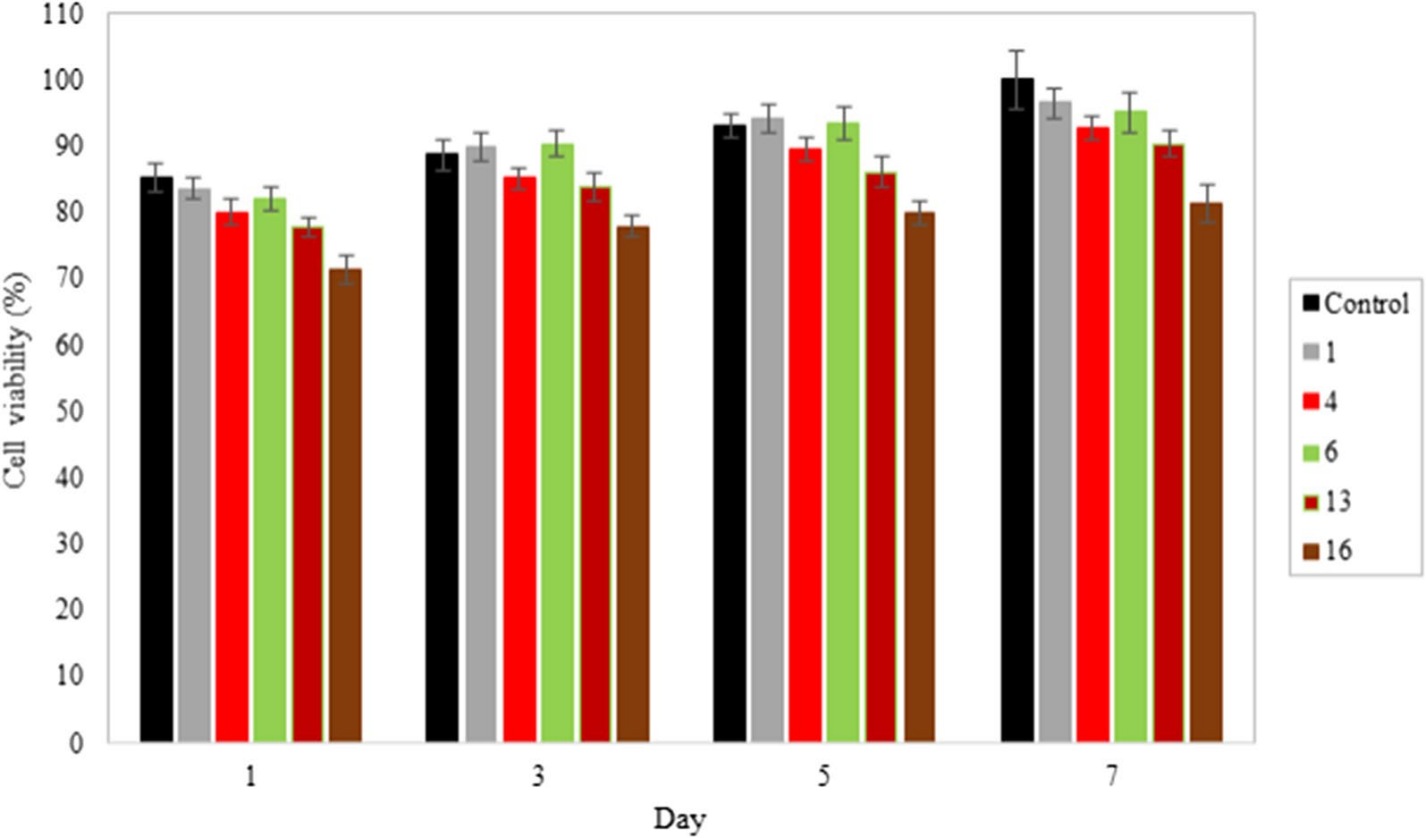
CLSM analysis of the biofilms formed by *S. aureus* (**a**, **b**, and **c**) and *P. aeruginosa* (**d**, **e**, and **f**), Control (**a** and **d**), nanocomposite in the absence of EMF (b and e), nanocomposite in the presence of EMF (**c** and **f**)

Visual observation of the effects of the nanocomposite and EMF application on biofilm inhibition was obtained using CLSM images (Fig. 19). Samples 6 and 16 were selected for biofilm inhibition of *S. aurous* and *P. aeruginosa*, respectively. The cells were stained with SYTO9 (green) for living cells and propidium iodide (red) for dead cells.

As shown in Fig. 19a, *S. aureus* bacteria adhere to the surface, enclosed in the extracellular polymeric substance, and form a dense and thick biofilm. After treatment of biofilm with sample 6, some of the bacteria are killed (Fig. 19b). In fact, AgNPs and IONPs, with a high surface-to-volume ratio, were released, dispersed, and bound to the bacteria, and damaged their wall, but the biofilm still existed and retained its overall matrix. By applying EMF, the diffusivity of the nanoparticles into the biofilm was increased, which led to a decrease in the biofilm density. A comparison of images (b) and (c) reveals that although the structure of sample 6 could not kill the bacteria of the biofilm effectively, nanoparticles inhibited the bacterial adhesion and suppressed EPS synthesis [9]. A similar trend was also observed for inhibition of *P. aeruginosa* biofilm. However, Ag/IO nanocomposites had a significant effect on *P. aeruginosa* biofilm compared to *S. aureus*. Figure 19d–f demonstrates that after treatment of biofilm with sample 16, the matrix is damaged and only some clusters of bacterial cells are retained. Furthermore, when the biofilm is exposed to EMF, more than 90% of the biofilm is eradicated and only a few bacterial microcolonies are survived. CLSM results are consistent with the results obtained from the crystal violet staining method.

Based on the outcome, it can be concluded that although silver is a strong antibacterial material, it has low diffusivity in biofilm. According to the scientific reports, using high concentrations of silver nanoparticles for further biofilm inhibition could not be considered an effective method due to the agglomeration and increase in the size of the nanoparticles. This fact is evident in the studies performed by Alharbi et al. (0.5%) [42], Cochis et al. (1.7–1.9%) [81], and Grumezescu et al. (1 mg/cm^2^) [82], that have used the same or even higher nanoparticle concentrations than the present study. On the other hand, the addition of IONPs to AgNPs and using EMF make the silver nanoparticles more functional and improve their efficiency for biofilm inhibition.

## Conclusion

In this research, various concentrations of AgNPs and IONPs were used in providing nanocomposites to evaluate their influence on the treatment of infected wounds. The results demonstrated that the use of IONPs can improve the effectiveness of the nanocompositesˈ antibacterial properties in such a way that the intended purpose was achieved without having to use high concentrations or doses of AgNPs, which are the main cause of the wound dressing's toxicity. Furthermore, EMF can have a superior effect on the release of the IONPs which can be followed by a remarkable release of the AgNPs, resulting in noticeable inhibition in biofilm formation to about 40%.

## Data Availability

The data that support the findings of this study are included within the article.

## Abbreviations

AgNPs: Silver nanoparticles
CLSM: Confocal laser scanning microscopy
DLS: Dynamic light scattering
DMEM: Dulbecco’s modified Eagle’s medium
DMF: Dimethylformamide
DMSO: Dimethyl sulfoxide
EDX: Energy-dispersive x-ray spectroscopy
ELISA: Enzyme-linked immunosorbent assay
EMF: External magnetic field
EPS: Extracellular polymeric substance
FBS: Fetal bovine serum
FE-SEM: Field emission- scanning electron microscopy
FTIR: Fourier-transform infrared spectroscopy
GA: Gum Arabic
IONPs: Iron oxide nanoparticles
MEF: Mouse embryonic fibroblast
MTT: 3-(4,5-Dimethylthiazol-2-yl)2,5-diphenyltetrazolium bromide
OD: Optical density
PBS: Phosphate-buffered saline
PCL: Polycaprolacton
PI: Propidium iodide
PVA: Polyvinyl alcohol
TEM: Transmission electron microscopy
TGA: Thermogravimetric analysis
VSM: Vibrating-sample magnetometer
XRD: X-ray powder diffraction
